# Update of species limits in the *Pristimantis myersi* clade (Anura: Craugastoridae), with the description of two new species from the western Andes of Ecuador

**DOI:** 10.7717/peerj.21075

**Published:** 2026-04-14

**Authors:** Mario H. Yánez-Muñoz, Mateo A. Vega-Yánez, Juan P. Reyes-Puig, Gabriela Lagla-Chimba, Christian Paucar-Veintimilla, Katherine Nicolalde-Tapia, Miguel Urgilés-Merchán, Julio C. Carrión-Olmedo, Carolina Reyes-Puig

**Affiliations:** 1 Unidad de Investigación, Instituto Nacional de Biodiversidad, Quito, Ecuador; 2 Fundación Oscar Efrén Reyes, Departamento de Ambiente, Baños, Ecuador; 3 Fundación EcoMinga, Red de Protección de Bosques Amenazados, Baños, Tungurahua, Ecuador; 4 Instituto de Biodiversidad Tropical, IBIOTROP, Museo de Zoología y Laboratorio de Zoología Terrestre, Colegio de Ciencias Biológicas y Ambientales, Universidad San Francisco de Quito, Quito, Pichincha, Ecuador

**Keywords:** Endemism, Cryptic diversity, Rain frogs, Taxonomy, Morphology

## Abstract

The tropical Andes represent not only one of the most biodiverse regions on the planet, but also a center of diversification and endemism for the hyperdiverse genus *Pristimantis*. Our study contributes to resolving and updating the taxonomy of one of the most diverse clades within the genus, the *Pristimantis myersi* clade. Here, we describe two new species of this clade and provide insights into their morphology, phylogenetic position, and biogeography. During field campaigns in 2022 and 2023, led by the Instituto Nacional de Biodiversidad in Cotacachi, Imbabura Province, Ecuador, specimens were collected. These specimens were then analyzed using an integrative approach that combined external morphology and DNA sequencing to describe the two new species and update the phylogenetic framework of the *P. myersi* clade. *Pristimantis cayapas* sp. nov. is a small species distinguished by narrow spatulate toes, tympanum present, a short snout, and distinctive reddish inguinal coloration. *Pristimantis dinardoi* sp. nov. is a small species characterized by a visible tympanum, digital pads of fingers and toes wider than the digits, a heel with a conical tubercle surrounded by subconical tubercles, and groins with a range of vivid hues. Both species occur in high montane evergreen forests of the North western Ecuadorian Andes. Genetic distances also support their recognition as new species, with values exceeding 2.5% for *P. dinardoi* and 4% for *P. cayapas* when compared with their closest relatives. Our findings expand the known diversity of the *P. myersi* clade in the northern Andes of Ecuador and highlight the importance of integrative taxonomic approaches in disentangling cryptic lineages.

## Introduction

Terrestrial frogs of the genus *Pristimantis* Jiménez de la Espada, 1870, clearly represent an outstanding model organism for research in systematics, taxonomy, evolution, phylogenetics, and biogeography (Mendoza et al., 2015; Zumel, Buckley & Ron, 2022; Yánez-Muñoz et al., 2025). Research on this group can facilitate understanding of the mechanisms driving speciation in small vertebrates of the Andes (Mendoza et al., 2015; Vásquez-Restrepo, Ribeiro-Júnior & Sánchez-Pacheco, 2024). In the last 15 years, significant progress has been made in understanding the cryptic diversity and evolutionary relationships of this highly speciose genus of anurans (Reyes-Puig & Mancero, 2022; Coloma & Duellman, 2024). Several studies have revealed substantial genetic and morphological diversity among Andean frog populations, highlighting the presence of cryptic species that are morphologically similar yet genetically distinct across this mountain system (*e.g*., Hutter & Guayasamin, 2015; Urgiles et al., 2019; Páez & Ron, 2019).

The tropical Andes are known as one of the most biodiverse regions in the world (Myers et al., 2000; Bax & Francesconi, 2019), with amphibians representing one of the most diverse groups within vertebrates (Hutter, Lambert & Wiens, 2017). Within the tropical Andes, *Pristimantis* not only exhibits the greatest species richness but is also among the most taxonomically dynamic amphibian groups, with many new species described over a relatively short time span (*e.g*., Guayasamin et al., 2015; Franco-Mena et al., 2023; Yánez-Muñoz et al., 2025). Many of these taxa have extremely restricted distributions, often limited by biogeographic barriers and/or elevation, underscoring both their evolutionary and biogeographic significance as well as their vulnerability to global threats (Ortega-Andrade et al., 2021; Kacoliris et al., 2022).

Recently, Franco-Mena et al. (2023), unveiled the evolutionary relationships and high cryptic diversity of one of the most dominant clades in the tropical Andes of Ecuador and Colombia, the *Pristimantis myersi* clade. Subsequent and parallel to this work, several lineages initially identified as candidate species have since been described, further refining our understanding of species diversity (Reyes-Puig et al., 2023; Yánez-Muñoz et al., 2025).

In this work we describe two new species of the genus *Pristimantis*, belonging to the subgenus *Trachyphrynus* and the *P. myersi* clade (Franco-Mena et al., 2023; Frost, 2025; Yánez-Muñoz et al., 2025). We provide a detailed account of their phylogenetic position and a morphological diagnosis highlighting the characters that distinguish them.

## Materials and Methods

### Ethics statement

This study fully adhered to the ARROW guidelines (Field et al., 2019). Ethical approval and authorization for fieldwork, collection, and handling of wild vertebrates were granted by the Ministry of Environment, Water, and Ecological Transition of Ecuador (MAATE), which is the legally designated authority for ethical oversight of wildlife research in Ecuador (Permit Nos. MAATE-ARSFC-2023-3346 and MAATE-ARSFC-2024-0847). All procedures complied with relevant national, international, and institutional guidelines on animal welfare and research ethics, including the Código Orgánico del Ambiente of Ecuador, and the ethical standards of the American Society of Ichthyologists and Herpetologists, the Herpetologists’ League, and the Society for the Study of Amphibians and Reptiles (Beaupre et al., 2004). We followed the 3R principles (Replacement, Reduction, and Refinement) by limiting sample sizes (24 adult individuals), excluding juveniles, and applying standardized, humane methods of handling, transport, and euthanasia to minimize stress and suffering (see below). For field research involving amphibians’ handling, we adhered to taxon-specific ethical recommendations for herpetological studies (Beaupre et al., 2004; American Veterinary Medical Association (AVMA), 2020), ensuring the responsible collection and conservation of the studied species.

We examined specimens deposited in the following herpetological collections (Appendix 1): Instituto Nacional de Biodiversidad (INABIO), Quito, Ecuador (DHMECN); Instituto Humboldt, Villa de Leyva, Colombia (IAvH, previously known as IND-AN); and Museo de Zoología, Universidad San Francisco de Quito, Quito, Ecuador (ZSFQ). Museum acronyms follow Frost (2025).

### Taxon sampling

For the taxonomic descriptions, we integrated multiple sources of evidence, including external morphological characters, linear morphometric variation, genetic and phylogenetic divergence, and geographic distributions. The new species were described based on a comprehensive approach that considered representative variation in the type series, observed both in life and in preservation. We have applied similar approaches to recognize and identify cryptic amphibian and reptile complexes in the north-western Ecuadorian Andes (Yánez-Muñoz et al., 2018, 2021; Reyes-Puig et al., 2020b, 2020a).

Family classification followed Barrientos et al. (2021), Motta et al. (2021) and Portik et al. (2023). Species recognition followed the unified species concept of De Queiroz (2005), which defines species as independently evolving metapopulation lineages supported by multiple lines of evidence. Morphological definitions, descriptive format, and terminology followed Duellman & Lehr (2009).

The electronic version of this article in Portable Document Format (PDF) will represent a published work according to the International Commission on Zoological Nomenclature (ICZN), and hence the new names contained in the electronic version are effectively published under that code from the electronic edition alone. This published work and the nomenclatural acts it contains have been registered in ZooBank, the online registration system for the ICZN. ZooBank LSIDs (Life Science Identifiers) can be resolved and the associated information viewed through any standard web browser by appending the LSID to the prefix http://zoobank.org/. The LSID for this publication is: **urn:lsid:zoobank.org:pub:8E8B82A4-3803-40D9-9CF4-9BB8C66B4B51**. The online version of this work is archived and available from the following digital repositories: PeerJ, PubMed Central SCIE and CLOCKSS.

### Field work

Herpetological surveys were conducted using visual encounter surveys (Rueda, Castro & Cortez, 2006), primarily during nocturnal hours. Fieldwork took place in the northwestern Andean slopes of Ecuador, specifically in the Cotacachi-Cayapas National Park, Imbabura Province, Ecuador, during expeditions in 2022–2023 organized by the Instituto Nacional de Biodiversidad (INABIO). A total of 24 individuals of two species were collected, which represents a limited sample relative to the number of individuals observed in the field. Juveniles were not collected in order to minimize impacts on populations.

Specimens were collected by hand, transported individually in plastic bags that contained a portion of moist vegetation to provide water, and maintained for no longer than 12 h until processing. During this period, animals were handled with care and monitored regularly to ensure adequate hydration and welfare. No additional feeding or enrichment was necessary due to the short holding period.

For euthanasia, benzocaine was applied topically using a spray applicator. The concentration used was 20% benzocaine solution, which is consistent with widely accepted and authorized protocols for amphibians (*e.g*., American Veterinary Medical Association (AVMA), 2020). No animals died prior to the initiation of the euthanasia procedure, and the criteria for euthanasia were applied consistently to all individuals collected. Following administration, death was confirmed by verifying the absence of vital signs (no heartbeat, no respiratory movements, response to firm toe pinch), in accordance with American Veterinary Medical Association (AVMA) (2020).

After euthanasia, a sample of muscle tissue was preserved in 95% ethanol for subsequent molecular analyses. Voucher specimens were fixed in 10% formalin and later preserved in 75% ethanol, following Rueda, Castro & Cortez (2006). All voucher specimens were deposited in scientific collections at the Instituto Nacional de Biodiversidad (INABIO), Ecuador.

### Morphological data

Taxonomic terminology follows Duellman & Lehr (2009). Morphometric measurements were taken with an electronic caliper (accuracy ±0.01 mm, rounded to 0.1 mm). The following morphological measurements were recorded according to Duellman & Lehr (2009) and subsequent descriptions (*e.g*., Yánez-Muñoz et al., 2025). We recorded the following morphometric variables: SVL = distance from snout tip to posterior margin of vent, HW = head width, HL = head length, ED = horizontal eye diameter, IOD = interorbital distance, EN = eye–nostril distance, TD = tympanic length, IND = inter-narinal distance, TL = tibia length, EW = upper eyelid width, FoL = foot length, HaL = hand length, F3D = disc width of finger III, and T4D = disc width of toe IV. Fingers were numbered pre-axially to post-axially from I–IV, and comparative toe lengths (I–V) were evaluated relative to toe IV. Sex, maturity, and reproductive condition were assessed through the presence of vocal slits, body size, and direct examination of gonads. Sexual dimorphism was analyzed descriptively by comparing SVL between sexes. Measurement definitions follow Yánez-Muñoz et al. (2025). Color in life was determined from field photographs provided by the original collectors. In the description of life and preserved coloration of the type series, colors are reported with the corresponding catalogue number from the (Köhler, 2012) standard in parentheses.

### Molecular data

DNA extraction and PCR amplification followed the methodology described by Reyes-Puig et al. (2024) and Yánez-Muñoz et al. (2025). Sequencing and bioinformatics procedures are described by Yánez-Muñoz et al. (2025). Bulk processing python script for FASTQ files processing, consensus generation, concatenation and FASTA files management is available in Zenodo by Carrión-Olmedo (2024).

Nanopore sequencing of partial mitogenomes flanking from 12S rRNA to ND1 region were performed at the Nucleic Acid Sequencing Laboratory of the Instituto Nacional de Biodiversidad (INABIO) in Quito, Ecuador.

A total of 35 partial mitogenomes were generated for specimens related to the *Pristimantis myersi* group, then they were imported to Mesquite (Maddison & Maddison, 2025). The character matrix was built with species in the *Trachyphrynus* subgenus *sensu* Bejarano-Muñoz et al. (2022), Franco-Mena et al. (2023), Reyes-Puig et al. (2023), Yánez-Muñoz et al. (2025), and other *Pristimantis* species from previous studies *i.e*., Darst & Cannatella (2004), Heinicke, Duellman & Hedges (2007), Hedges, Duellman & Heinicke (2008), Heinicke et al. (2018), García-R et al. (2012), Barrio-Amoros, Heinicke & Hedges (2013), Guayasamin et al. (2015), Rivera-Correa & Daza (2016), Székely et al. (2016), Gonzalez-Duran et al. (2017), Pintanel et al. (2019), Bejarano-Muñoz et al. (2022), Zumel, Buckley & Ron (2022), and Means et al. (2023).

Phylogenetic analyses were performed on a partitioned dataset comprising 12S rRNA, tRNA-Val, 16S rRNA, tRNA-Leu, and the codon positions of ND1. The matrix was aligned using the default parameters of MAFFT (Katoh & Standley, 2013) and subsequently inspected visually to identify any unambiguous alignment errors. When short, unambiguous misalignments (typically 1–5 bp shifts affecting positional homology) were detected, the affected fragments were locally re-aligned using MAFFT and minimally adjusted by hand to correct gap placement.

Substitution model evaluation and maximum likelihood tree inference were performed using IQ-TREE (Trifinopoulos et al., 2016) under default settings. Support values consisted of 2,000 UF (Ultrafast) bootstraps and SH-aLRT (Shimodaira–Hasegawa approximate likelihood ratio) test with 1,000 replicates (Guindon et al., 2010). Support values mentioned herein follow this format: SH-aLRT support (%)/ultrafast bootstrap support (%).

To estimate uncorrected pairwise genetic p-distances, we used a 921 bp subset of the 16S rRNA fragment. We selected a 16S rRNA partial fragment as it was the length for which completeness was consistent across both our samples and publicly available sequences. Using a single, uniformly represented locus avoids biases associated with missing data, even when applying pairwise deletion in distance calculations. P-distances were computed in Molecular Evolutionary Genetics Analysis (MEGA) version 11 (Tamura, Stecher & Kumar, 2021). The obtained distance matrix was plotted as a heat map in R 4.2.1 (R Core Team, 2023), with the package *dplyr* (Wickham et al., 2023) package.

## Results

**Figure 1:**
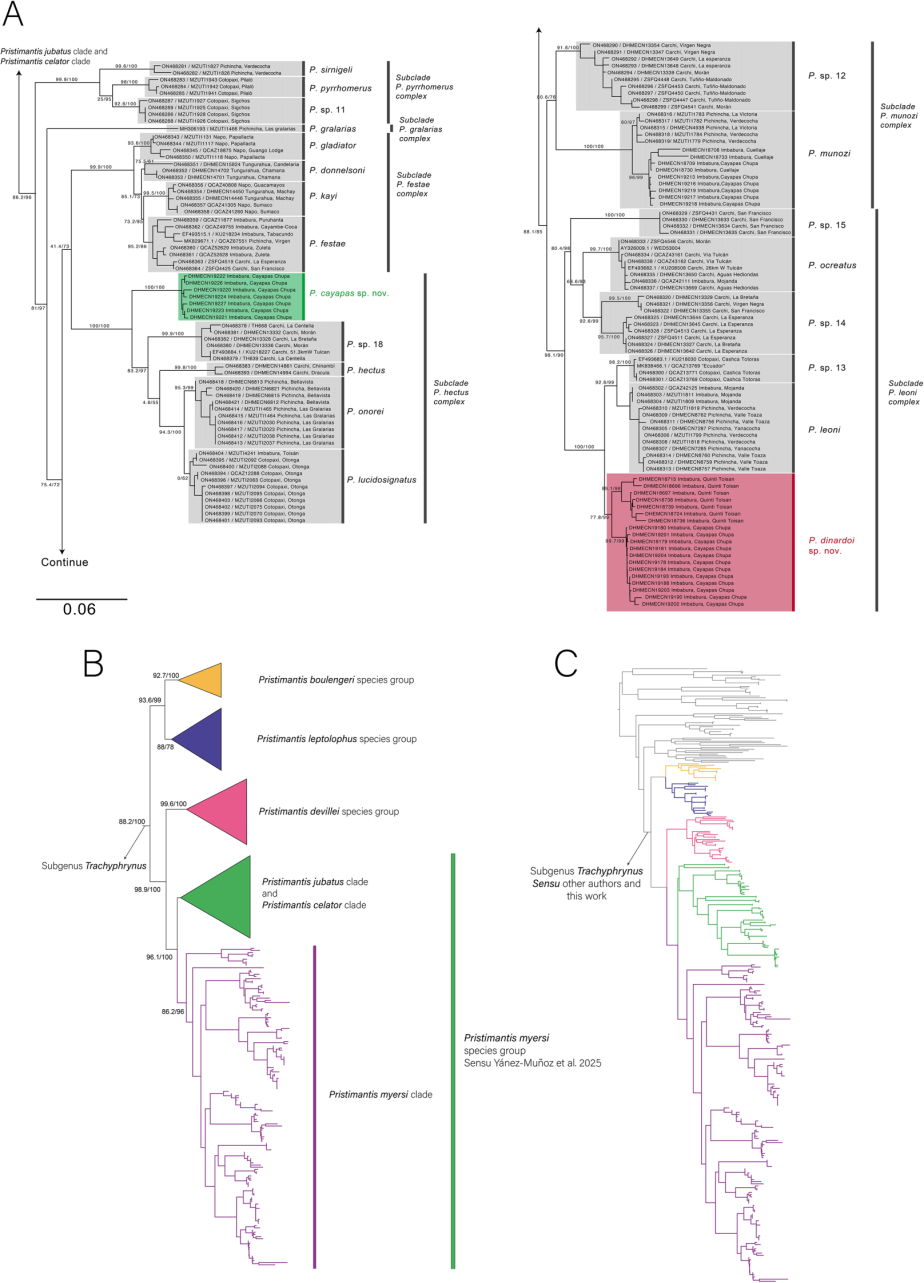
Phylogeny of *Pristimantis myersi* clade. (A) Maximum Likelihood phylogenetic tree obtained from 3,955 bp long for 146 individuals. (B) Relationship of *Pristimantis myersi* clade to *P. celator* clade, *Pristimantis jubatus* clade and others in the subgenus *Trachryphrynus*; (C) Phylogenetic position of the *Pristimantis myersi* clade and sister clades within the genus *Pristimantis*, from 330 individuals.

**Phylogenetic relationships and genetic distances** (Fig 1, Supplemental Fig. SM1.1). The matrix comprises 3.8 kbp of aligned sequences for 314 individuals. The selected substitution models are as follows: TIM2+F+I+G4 for 12S rRNA, TIM2e+G4 for tRNA-Val, TIM2+F+I+G4 for 16S rRNA, HKY+F+G4 for tRNA-Leu, TIM2e+G4 for ND1 first position, TPM3+F+I for ND1 second position, TN+F for ND1 third position. Full partition model BIC score: 121910.725 (LnL: -58214.618 df:663).

Within the *Pristimantis myersi* group, our phylogenies among species are consistent with those from Franco-Mena et al. (2023) and Reyes-Puig et al. (2023). However, we recovered two new lineages, herein described as new.

We found six highly supported clades within the *Pristimantis myersi* clade (Fig. 1). Support values are reported as SH-aLRT (%)/ultrafast bootstrap (%). These include:

(1) Subclade *P. pyrrhomerus* complex (bootstrap 99.9/100) composed of: *P. sirnigeli* Yánez-Muñoz et al., 2010*+P. pyrrhomerus* (Lynch, 1976) + *P*. sp. 11.
(2) Subclade *P. gralarias* complex (bootstrap 81/97) composed of: *P. gralarias* Guayasamin, Arteaga & Hutter, 2018.
(3) Subclade *P. festae* complex (bootstrap 99.9/100) composed of: *P. gladiator* (Lynch, 1976) *+ P. donnelsoni* Reyes-Puig et al., 2023*+P. kayi* Reyes-Puig et al., 2023*+ P. festae* (Peracca, 1904).
(4) Subclade *P. hectus* complex (bootstrap 100/100) composed of (*P. cayapas* sp. nov. *+ P*. sp. 18 *+ P. hectus* (Lynch & Burrowes, 1990) *+ P. onorei* Rodder & Schmitz, 2009 *+ P. lucidosignatus* Rodder & Schmitz, 2009.
(5) Subclade *P. munozi* complex (bootstrap 80.6/76) composed of: *P*.sp 12*+P. munozi*, 2014.
(6) Subclade *P. leoni* complex (bootstrap 100/100) composed of: *P*. sp. 15 *+ P. ocreatus* (Lynch, 1976) *+ P*. sp. 14+*P*. sp. 13+ *P. leoni* (Lynch, 1976) + *P. dinardoi* sp. nov.

The new species described here (*Pristimantis cayapas* sp. nov. and *P. dinardoi* sp. nov.) nest with their sister species respectively in the subclades *Pristimantis hectus* complex and *P. leoni* complex. During the barcoding-based identification of new *Pristimantis* collections from Imbabura, we also recorded additional localities for *P. munozi*, a species previously known only from Pichincha Province. *Pristimantis* These new localities in Cayapas-Chupa and Cuellaje, Imbabura Province represent the northernmost records of *P. munozi* expanding 70 km to the north its known distribution. The genetic distances between the *sensu stricto* populations of *Pristimantis munozi* and the populations of Imbabura province reached 1.3% genetic differentiation.

The genetic distance of *Pristimantis cayapas* sp. nov. is greater than 4.00% among congeners of the *P. hectus* complex, while the genetic distance of *P. dinardoi* sp. nov. is greater than 2.50% among congeners of the *P. leoni* complex.

**New species**

***Pristimantis cayapas*** **sp. nov.**

**LSIDurn:lsid:zoobank.org:act:82585CA0-70C5-49DA-81ED-125677E57A9F**

***Common name in Spanish: Cutín Cayapa***

***Suggested common English name: Cayapa´s rainfrog***

**Figure 2:**
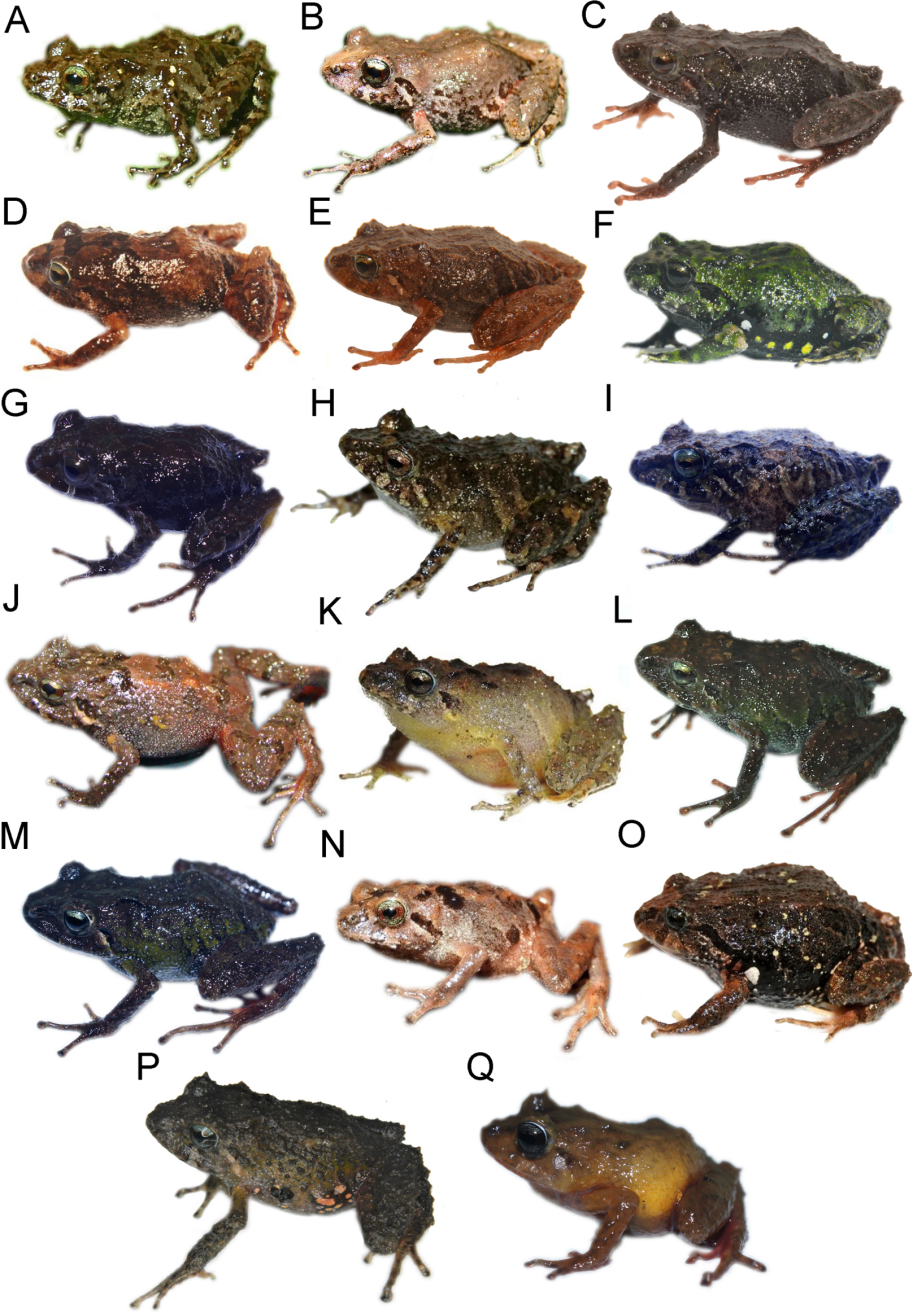
*Pristimantis* (*Trachyphrynus*) *myersi* clade from Ecuador. Subclade *P. pyrrhomerus* complex: (A) *P. sirnigeli*, male Holotype (DHMECN 06803), Cordillera de Saloya, Pichincha Prov.; (B) P. *pyrrhomerus*, female (Field series ANF-1139), Peñas Coloradas, Cotopaxi Prov. Subclade *P. festae* complex: (C) *P. gladiator*, female (DHMECN 12663), Guango Lodge, Napo Prov.; (D) *P. donnelsoni*, male (no collected specimen), Volcan Tungurahua, Tungurahua Prov.; (E) *P. kayi*, female paratype (DHMECN 14446) Reserva Machay, Tungurauna, Prov.; (F) *P. festae*, female (DHMECN 16833) Laguna de mojanda, Pichincha Prov.). Subclade *P. hectus* complex: (G) *P. cayapas* sp. nov., female Holotype (DHMECN 19220), Cordillera Cayapas Chupas, Imbabura; (H) *P*. sp. 18, female (DHMECN 13332), Morán, Carchi Prov.; (I) *P. hectus*, female (DHMECN 19494), Reserva Dracula, Carchi Prov.; (J) *P. onorei*, female (DHMECN 6813), Reserva Bellavista, Pichincha Prov. Subclade *P. floridus* complex: (K) *P. sp*.12, male (DHMECN 13647), Virgen Negra, Carchi Prov.; (L) *P. munozi*, female, (DHMECN 4938), Reserva Verde Cocha, Pichincha Prov. Subclade *P. leoni* complex: (M) *P*. sp. 15, female (DHMECN 13635), San Fancisco de Pioter, Carchi Prov.; (N) *P*. sp. 14, female (DHMECN 13644), El Chamizo, Carchi Prov.; (O) *P. ocreatus, female* (DHMECN 13655), Carchi Prov.; (P) *P. leoni*, female (DHMECN 16832), Mojanda, Pichincha Prov.; (Q) *P. dinardoi* sp. nov., female Holotype (DHMECN 19202). Photographs: All photographic by Mario H. Yánez-Muñoz, except: (2) Fernando Rojas, (8, 11, 13, 14, 15) Diego Batallas-Revelo.

**Figure 3:**
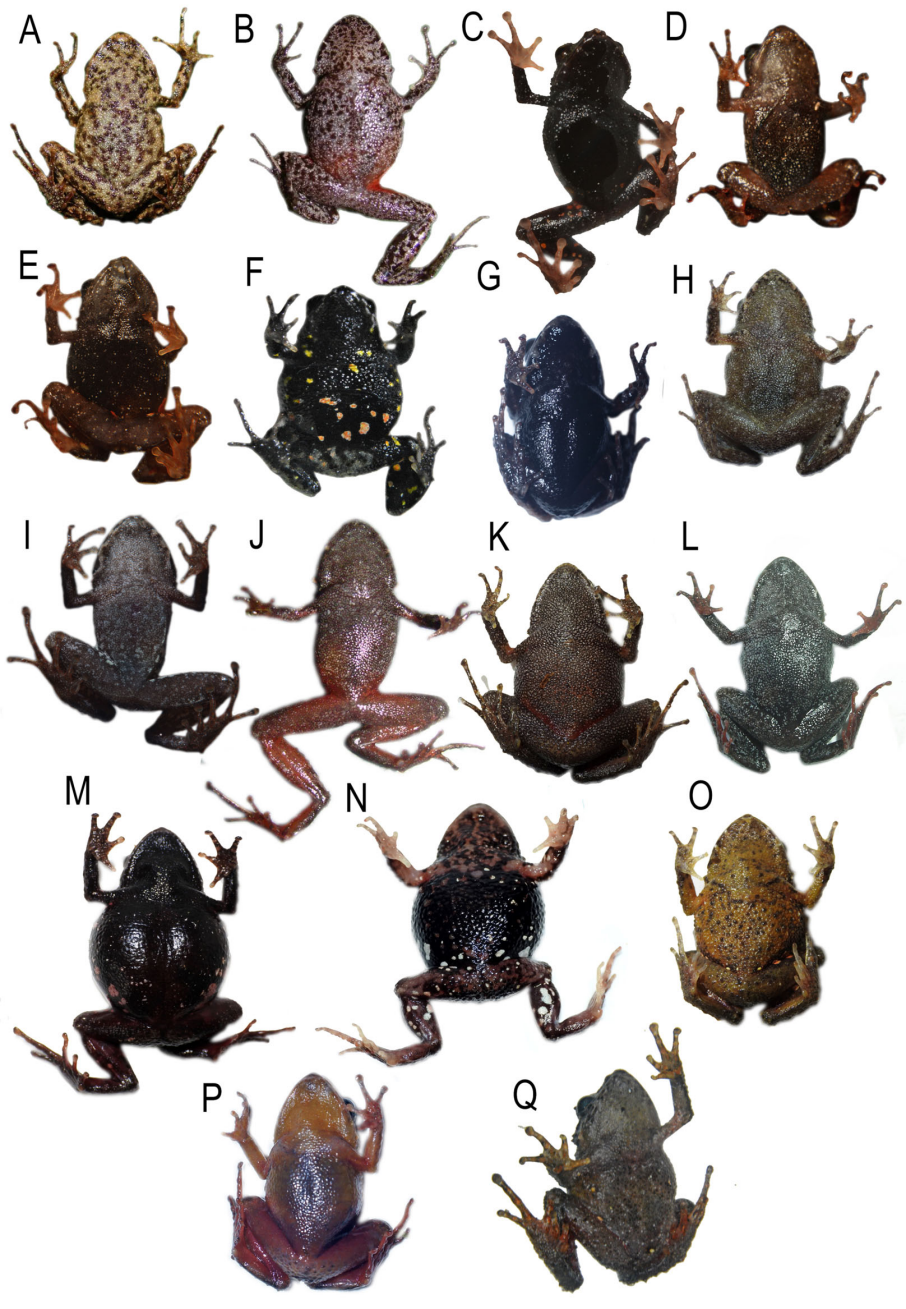
Detail of ventral view de *Pristimantis* (*Trachyphrynus*) *myersi* clade from Ecuador. Subclade *P. pyrrhomerus*: (A) *P. sirnigeli*, male Holotype (DHMECN 06803); (B) P. *pyrrhomerus*, female (Field series ANF-1139). Subclade *P. festae* complex: (C) *P. gladiator*, female (DHMECN 12663); (D) *P. donnelsoni*, male (no collected specimen); (E) *P. kayi*, female paratype (DHMECN 14446); (F) *P. festae*, female (DHMECN 16833). Subclade *P. hectus* complex: (G) *P. cayapas *sp. nov., female Holotype (DHMECN 19220); (H) *P*. sp. 18, female (DHMECN 13332); (I) *P. hectus*, female (DHMECN 19494); (J) *P. onorei*, female (DHMECN 6813). Subclade *P. floridus* complex: (K) *P. sp*. 12, male (DHMECN 13647); (L) *P. munozi*, female, (DHMECN 4938). Subclade *P. leoni* complex: (M) *P*. sp. 15, female (DHMECN 13635); (N) *P. ocreatus, female* (DHMECN 13655), (O) *P*. sp. 14, female (DHMECN 13644); (P) *P. dinardoi* sp. nov., female Holotype (DHMECN 19202), (Q) *P. leoni*, female (DHMECN 16832). Photographs: All photographic by Mario H. Yánez-Muñoz, except: (A, B) Fernando Rojas (H, K, M, N,O), Diego Batallas-Revelo.

**Figure 4:**
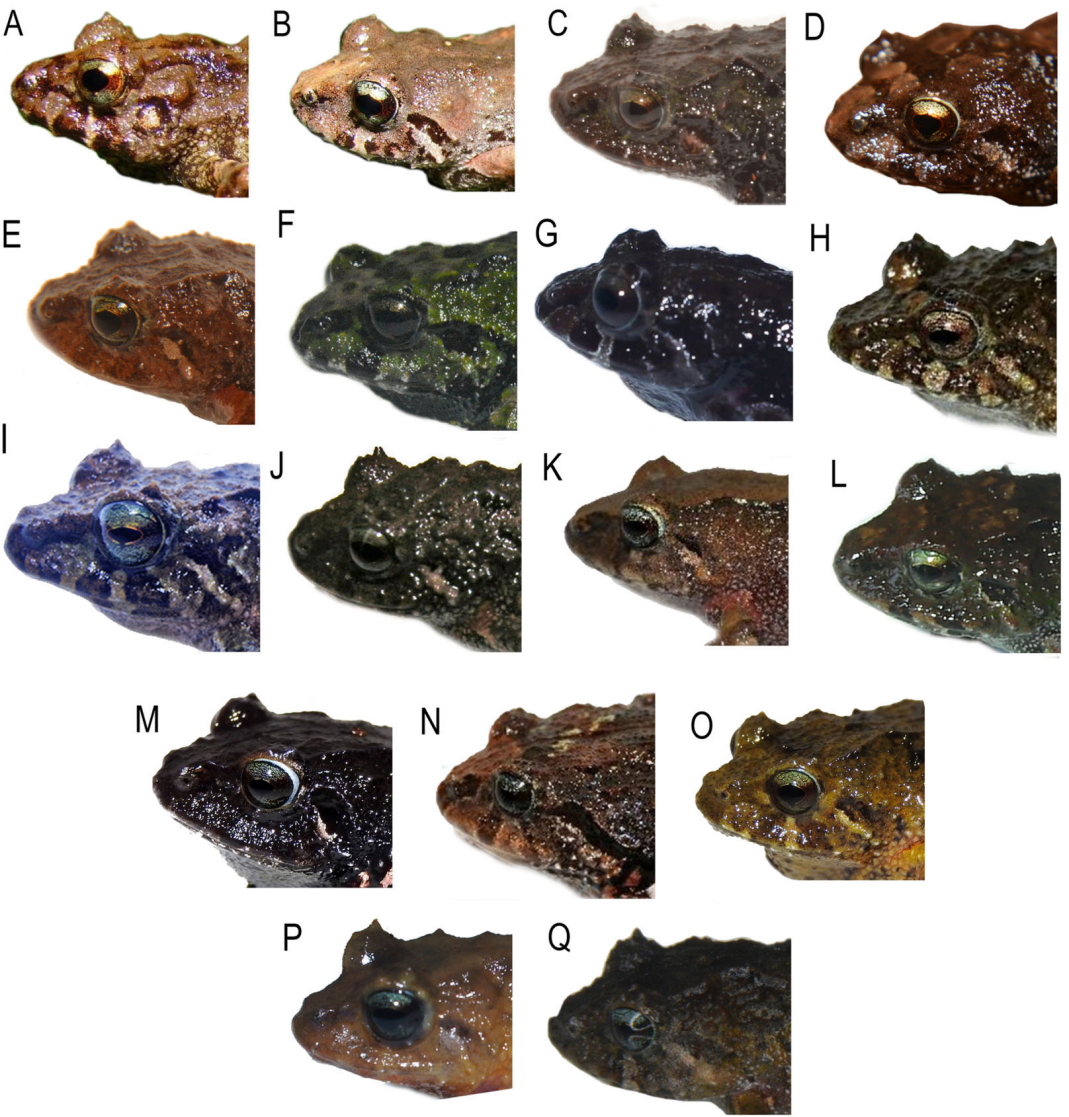
Detail of the heads in profile view de *Pristimantis* (*Trachyphrynus*) *myersi* clade from Ecuador. Subclade *P. pyrrhomerus*: (A) *P. sirnigeli*, male Holotype (DHMECN 06803); (B) P. *pyrrhomerus*, female (Field series ANF-1139). Subclade *P. festae* complex: (C) *P. gladiator*, female (DHMECN 12663); (D) *P. donnelsoni*, male (no collected specimen); (E) *P. kayi*, female paratype (DHMECN 14446); (F) *P. festae*, female (DHMECN 16833). Subclade *P. hectus* complex: (G) *P. cayapas* sp. nov., female Holotype (DHMECN 19220); (H) *P*. sp. 18, female (DHMECN 13332); (I) *P. hectus*, female (DHMECN 19494); (J) *P. onorei*, female (DHMECN 6813). Subclade *P. floridus* complex: (K) *P. sp*.12, male (DHMECN 13647); (L) *P. munozi*, female, (DHMECN 4938). Subclade *P. leoni* complex: (M) *P*. sp. 15, female (DHMECN 13635); (N) *P. ocreatus, female* (DHMECN 13655), (O) *P*. sp. 14, female (DHMECN 13644); (P) *P. dinardoi* sp. nov., female Holotype (DHMECN 19202), (Q) *P. leoni*, female (DHMECN 16832). Photographs: All photographic by Mario H. Yánez-Muñoz, except: (A, B) Fernando Rojas (H, K, M, N, O), Diego Batallas-Revelo.

**Figure 5:**
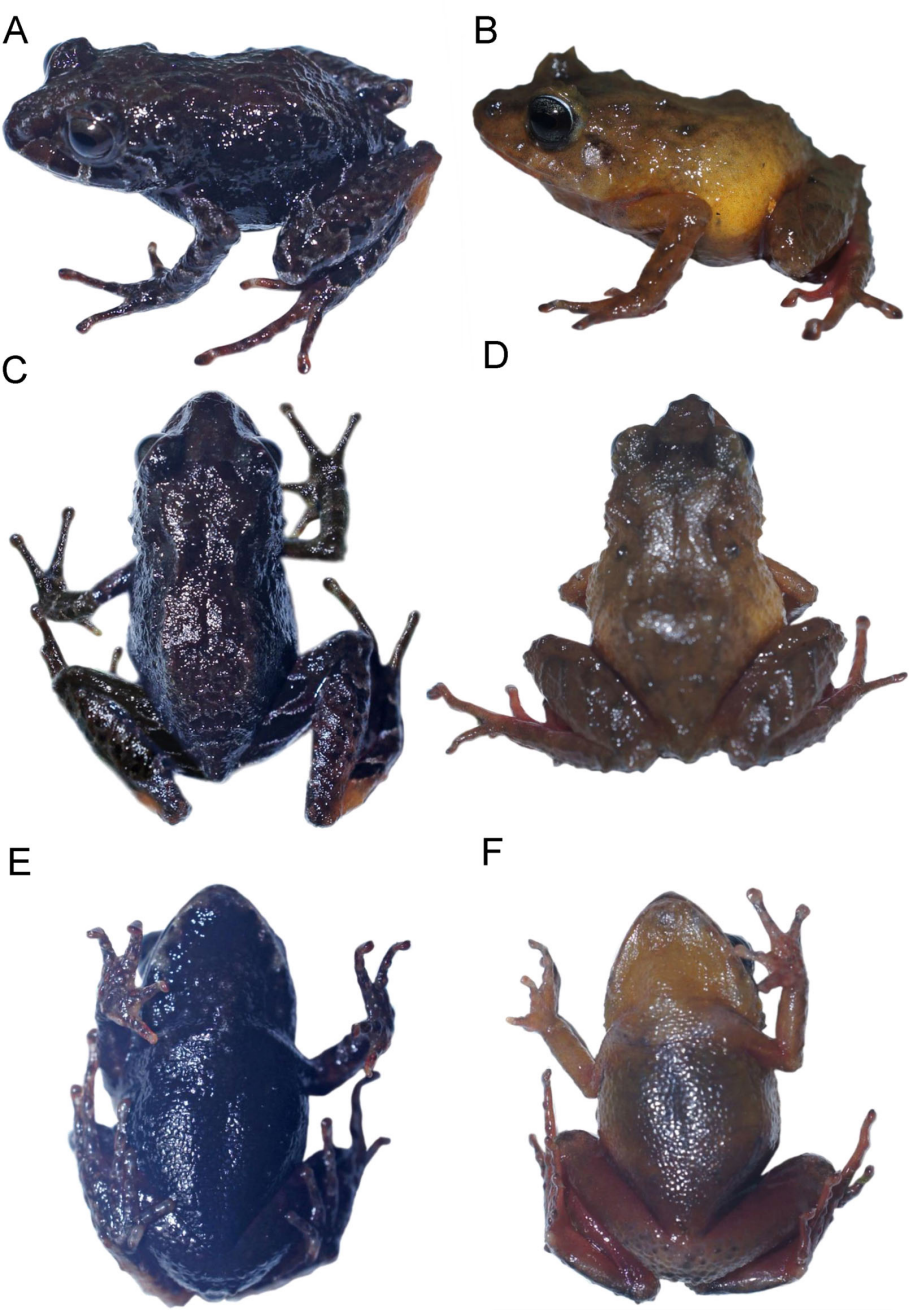
Two new species of western ecuadorian Andes. Photographs in life. (A, C, E) *Pristimantis cayapas* sp. nov., holotype DHMECN 19220. (B, D, F) *Pristimantis dinardoi* sp. nov., holotype DHMECN 19202.

**Figure 6:**
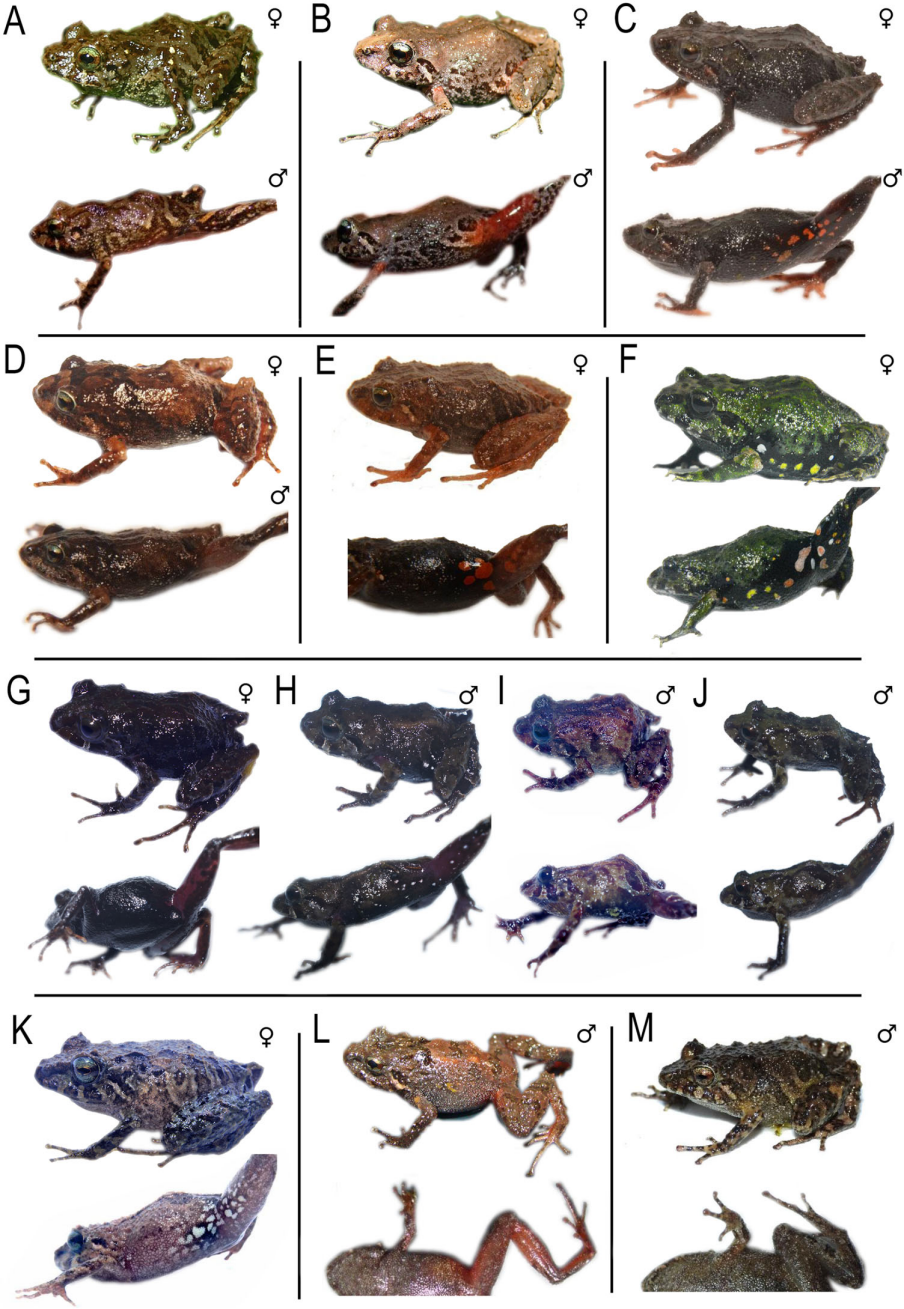
Comparison *Pristimantis* (*Trachyphrynus*) *myersi* clade from Ecuador. Subclade *P. pyrrhomerus* complex: (A) *P. sirnigeli*, male Holotype (DHMECN 06803); (B) *P. pyrrhomerus*, female (Field series ANF-1139). Subclade *P. festae* complex: (C) *P. gladiator*, female (DHMECN 12663); (D) *P. donnelsoni*, male (no collected specimen); (E) *P. kayi*, female paratype (DHMECN 14446); (F) *P. festae*, female (DHMECN 16833). Subclade *P. hectus* complex: (G–J) *P. cayapas* sp.nov., (G) female Holotype (DHMECN 19220), (H) female paratype (DHMECN 19222), (I) male paratype (DHMECN, 19221), (J) male (DHMECN 19224); (K) *P. hectus*, female (DHMECN 19494); (L) *P. onorei*, female (DHMECN 6813); (M) *P*. sp. 18, female (DHMECN 13332). All photographic by Mario H. Yánez-Muñoz, except: (B) Fernando Rojas, (M) Diego Batallas-Revelo.

**Figure 7:**
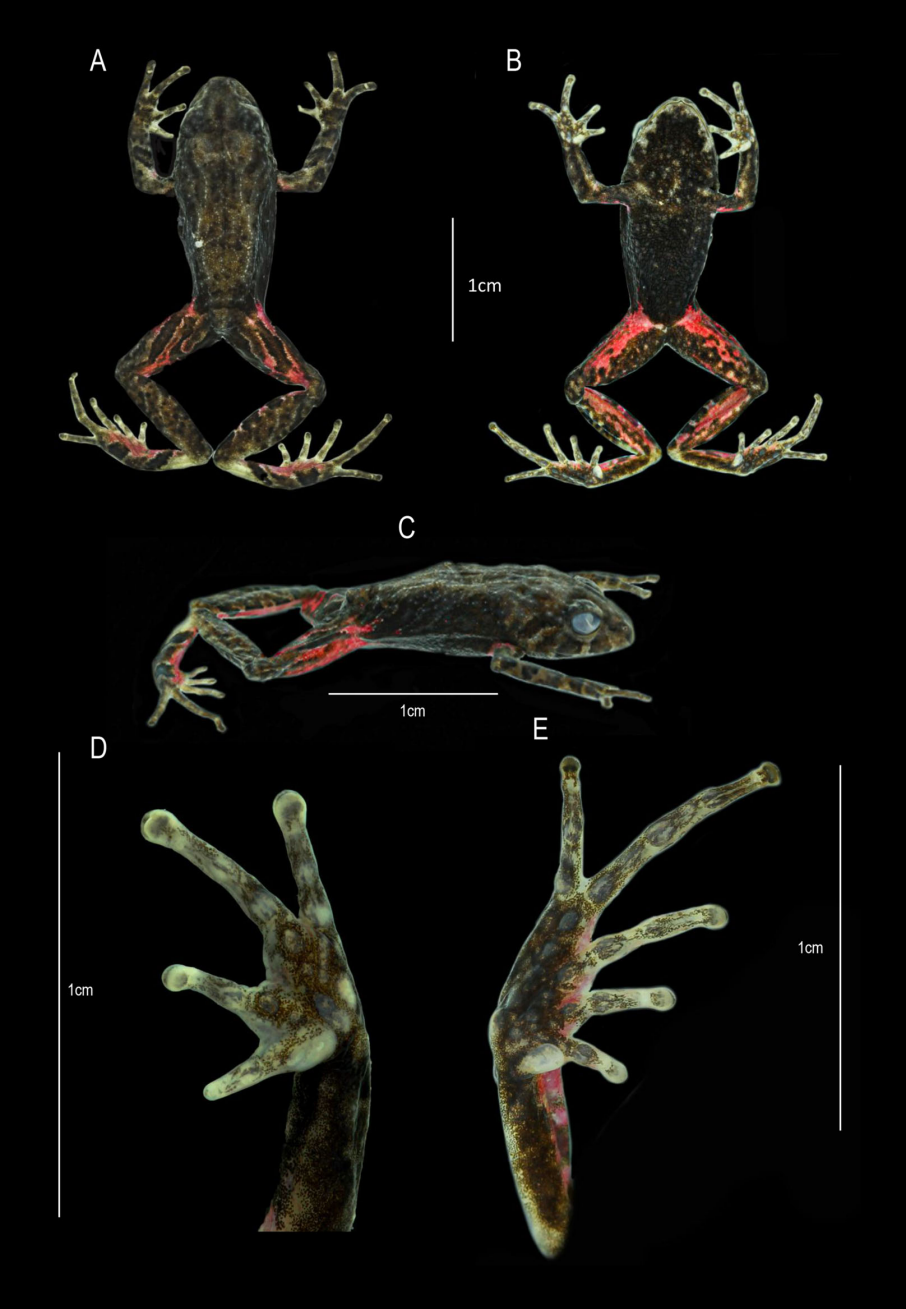
*Pristimantis cayapas* sp. nov., holotype DHMECN 19220, adult female from Cotacachi, Imbabura Province, Ecuador. (A) Dorsal view. (B) Ventral view. (C) Lateral view of body. (D) Palmar view of right hand. (E) Plantar view of right foot. Scale bars = 1 cm. Photographs by: Katherine Nicolalde-Tapia.

**Holotype (**Figs. 2–7**)**. DHMECN 19220, adult female, Cayapas-Chupa, Piñán, Parque Nacional Cotacachi-Cayapas, 6 de Julio de Cuellaje parish, Cotacachi canton, Imbabura province, Ecuador, (0,53813, −78,48255; 3,290 m), collected on 27 September 2023 by Mario Humberto Yánez-Muñoz, Jorge Brito and Jorge Páez.

**Paratype (**Supplemental Fig. SM1.2, 1♀, 4♂**)**. A total of five specimens were collected in the same type locality and data collectors. Adult female (1): DHMECN 19222 (0,53869, −78,48268; 3,310 m) on 28 September 2023. Adult males (3): DHMECN 19221, with the same data of Holotype; DHMECN 19223 (0,53869, −78,48268; 3,310 m) collected on 29 September 2023 and DHMECN 19224, (0,53964, −78,48306; 3,260 m) collected on 30 September 2023; DHMECN 19226 (0,53869, −78,48268; 3,310) collected on 2 October 2023. Subadult males (2): DHMECN 19227 (0,532153, −78,477099; 3,410 m) collected on 2 October 2023.

**Diagnosis.** *Pristimantis cayapas* sp. nov. is a member of the *Pristimantis myersi* species group and clade, characterized by the following combination of characters: (1) dorsal skin shagreen with occipital, scapular, dorsolateral and postero-sacral thin dermal ridges; flanks aereolate with scattered tubercles; discoidal fold absent, coarsely areolate skin on venter; (2) tympanum present, visible, tympanic annulus clearly defined, round, with a thick supratympanic fold, horizontal diameter of tympanum equal to 45.83% of eye diameter (*n* = 7), rounded postrictal tubercles present; (3) snout short, curved anteroventrally in profile, subacuminate in dorsal view; (4) upper eyelid with rounded tubercles; cranial crest absent; (5) dentigerous processes of vomers positioned posterior to level of choanae, finely oblique in posterior outline, barely visible until absent; (6) males with vocal sacs present, vocal slits absent, nuptial pads absent; (7) finger I shorter than finger II; digits with narrow discs, digital pads as wide as the digit; (8) fingers with lateral fringes defined, basally pronounced; (9) ulnar tubercles present, rounded low on the outer edge of the ulna; (10) heel and outer edge of the tarsus with two and three rounded to subconical tubercles; inner tarsal fold absent; (11) two metatarsal tubercles, external small subconical, internal oval, two times larger than the external metatarsal; supernumerary tubercles low and rounded; (12) toes with weakly defined lateral fringes, basally pronounced, interdigital membrane absent; (13) dorsum in females cinnamon brown to yellowish brown, with dark brown flanks (finely speckled with white in life), dermal ridges finely outlined in white or cream (light brown to greenish brown in life), transversal bars on dorsal surfaces of limbs, supratympanic, canthal and supralabial bars (finely outlined with white in life), with interspaces cinnamon brown or yellowish brown with pink (puppy red in life), belly homogeneously dark brown to greyish brown, with hidden surfaces of thighs and groin pink (puppy red in life). Dorsum in males dorsally and flanks yellowish brown, greyish brown, dark brown bars on forelimbs and hind limbs, belly in greyish brown background with irregular dark marks. Iris chocolate brown to golden brown; (14) adults small, males SVL = 12.38–13.9 mm (mean = 13.14 mm, *n* = 5), females SVL = 17.29–18.21 mm (mean = 17.75 mm, *n* = 2).

**Comparison with other species** (Figs. 2–4, 6**).** The new species differs from its close relatives (*P. hectus*, *P. onorei*, *P. lucidosignatus*, and *P*. sp. 18; Fig. 1) by its small body size, narrow toes (*vs*. flared and spatulate), short snout (long and angular in congeners), and distinctive inguinal coloration, with the posterior surfaces of the thighs and legs being wine-red (Fig. 2). *Pristimantis hectus* is readily distinguished by its expanded, lanceolate digital pads (Lynch & Burrowes, 1990), which are absent in *P. cayapas*. In addition, *P. cayapas* exhibits red coloration on the groin and hidden surfaces of the thighs, a trait absent in *P. hectus* (Lynch & Burrowes, 1990). The tympanum in *P. cayapas* is present and visible, with a clearly defined tympanic annulus, whereas it is completely concealed beneath the skin in females of *P. onorei* (Rodder & Schmitz, 2009). The upper eyelids bear conical tubercles in *P. onorei* and *P. lucidosignatus*, but rounded tubercles in *P. cayapas*. Finally, the anterior and posterior surfaces of the thighs are uniformly light brown in *P. onorei* and *P. lucidosignatus* (Rodder & Schmitz, 2009), but puppy-red in *P. cayapas*.

Genetic distances between *Pristimantis cayapas* and its closely related species (*P*. sp. 18, *P. hectus*, *P. onorei*, and *P. lucidosignatus*) are 4.43%, 4.24%, 4.55%, and 4.01%, respectively. Genetic distances with other species in the clade range from 6.51% to 12.49% (Supplemental Fig. SM1.1).

**Description of the holotype (**Figs. 5, 6**).** Adult female (DHMECN 19220), head longer than wide. Snout short, curved anteroventrally in profile, subacuminate in dorsal view with low rounded; eye-nostril distance 10.71% of SVL, canthus rostralis in cross section rounded, in dorsal view concave, loreal region concave, protruding nares directed dorsolaterally; interorbital area with flat, interorbital distance 60.65% wider than upper eyelid; cranial crest absent, with occipital and scapular thin dermal ridges; upper eyelid with two or three rounded tubercles; tympanum evident, tympanic annulus clearly defined, round, with a thick supratympanic fold, horizontal diameter of tympanum equal to 40.10% of eye diameter, rounded postrictal tubercles present; choanae small, oval in outline, not covered by palatal floor of maxilla; dentigerous processes of vomers positioned posterior to level of choanae, finely oblique in posterior outline, barely visible, no visible teeth, tongue longer than wide, ovoid, 40% of length of tongue attached to the floor of the mouth.

Texture of dorsum shagreen with dorsolateral and postero-sacral thin dermal ridges; with several minute tubercles aggregated in the dorsal surface of the sacral region, flanks areolate with dispersed tubercles; discoidal fold absent, venter areolate with tiny scattered tubercles; ulnar tubercles present, rounded and low on the outer edge of the ulna; outer palmar tubercle in divided “U”-shaped, approximately the same size as oval outer thenar tubercle; supernumerary tubercles present and flattened; subarticular tubercles defined, flattened in lateral view. Fingers with lateral fringes present, pronounced at the base of digits, discs the same width as the digits. Hind limbs slender (TL 50.79% of SVL; FL 51.78% of SVL); heel and outer edge of the tarsus with two and three rounded tubercles, inner tarsal fold absent; two metatarsal tubercles, external small subconical, internal oval two times larger than the external metatarsal; supernumerary tubercles low rounded; toes with weakly defined lateral fringes, basally pronounced, interdigital membrane absent; toe V is longer than toe III but does not extend to the distal subarticular tubercle of toe IV.

**Holotype coloration in preservative (**Fig. 7**)**. Colors were standardized following Köhler (2012). Dorsum in shades of raw umber (22) to antique brown (24) with scattered small irregular sepia markings (279) and finely delineated with whitish lime green (111). Interorbital region, prefrontal and eyelids greyish olive (274). Canthal, labial and supratympanic markings of sepia color (286). Labial markings are separated by olive horn-colored interspaces (16). Upper tympanic marking with finely delineated edges of pale lime green (112). Flanks and belly vandyke brown (281). Throat dusky brown (285), with markings on the edge of the lower jaw pale lime green (112). Axillae, groin, and anterior surfaces of the hind legs with distinctive spectrum-red markings (67). Rear surfaces of thighs with bands of vandike brown (281) with medium olive brown markings (278).

**Holotype coloration in life (**Fig. 5**).** Dorsal coloration dark carmine (61), with distinctive interoribal bar dark bluish purple (230), dark pearl gray (290) spots on eyelid and occipital region. Dusky brown (285) flanks and belly. Flanks with finely dotted lines with smoky white (261). Marron (39) canthal, supralabial and supratimpanic bars, separated by interspaces delineated by smoky white (261). Hands, forearms, legs and thighs, outlined in smoky white (261). Posterior surfaces of thighs, with bands sepia (286), finely outlined in smoky white (261) with interspaces in various tones from spinel pink (235) to rose (234). Dorsal surfaces of the leg in sepia (279) with dusky brown (285) bars. Heel base medium chrome orange (75). Axillae and groin and anterior thigh surfaces rose (234) and spinel pink (235). Iris mikado brown (42) with mid row number (280) with fine black reticulations.

**Measurements (in mm) of holotype.** SVL = 18.21; HW = 6.30; HL = 6.43; ED = 2.22; IOD = 2.16; EN = 1.95; TD = 0.89; IND = 1.76; TL = 9.25; EW = 1.31; FoL = 9.43; HaL = 5.48; FW = 0.65; TW = 0.63.

**Variation**. *Pristimantis cayapas* sp. nov. shows sexual dimorphism in body size (Supplemental Fig. SM1.2) and coloration. Males of this species are between 0.6 and 0.8 times smaller in SVL than females (Supplemental Table SM2.1). Other morphometric variation in the type series is shown in Supplemental Table SM2.1.

In life dorsal coloration varies from fuscous (283), as in specimens DHMECN 19222 (H) and DHMECN 19224 (J), to ultramarine blue (198) to smalt blue (184), as in DHMECN 19221 (I). Some individuals, such as DHMECN 19222 (H) and DHMECN 19224 (J), exhibit an interorbital bar of dark bluish purple (230), paler than that of the holotype; whereas in others, such as DHMECN 19221 (I), this bar is barely distinguishable, with a tone similar to ultramarine blue (198). The upper eyelid and occipital region may resemble the holotype, with coloration ranging from glaucous (289) to dark pearl gray (290), as in DHMECN 19222 (H) and DHMECN 19224 (J), or may be scarcely visible, as in DHMECN 19221 (I). Flanks display a color range from a dusky brown (285) background with oblique olive-gray (265) to light drab (269) bands, lightly edged with smoky white (261), as in DHMECN 19222 (H) and DHMECN 19224 (J); to flanks colored light bluish purple (228) with large smoke gray (266) blotches and areas dotted with smoky white (261), as in DHMECN 19221 (I). The ventral region varies from dusky brown (285), as in DHMECN 19222 (H) and DHMECN 19224 (J), to blue black (187), as in DHMECN 19221 (I). The canthal, supralabial, and supratympanic bars range from maroon (39) or warm sepia (40), separated by olive-gray (265) spaces edged with smoky white (261), as in DHMECN 19222 (H) and DHMECN 19224 (J); to bars varying between ultramarine (182) and smalt blue (185), separated by bluish purple (229) to dark bluish purple (230) spaces, bordered by small spots of smoky white (161), as in DHMECN 19221 (I). The dorsal surfaces of the legs show variable coloration, including sepia (279) and dusky brown (285) tones with brownish olive (292) bars, as in DHMECN 19222 (H) and DHMECN 19224 (J), as well as blue black (187) with shades of bluish purple (229) and interspaces of smoke gray (267), as in DHMECN 19221 (I). The heel base may or may not exhibit light orange tones. In some specimens, such as DHMECN 19224 (J), these areas lack distinctive coloration. The iris shows consistent coloration across all specimens, matching that of the holotype.

Variation in preservative (Supplemental Fig. SM1.2). The specimens examined show notable variation in preserved coloration. This variation is evident in the intensity, distribution, and contrast of chromatic patterns present on the dorsum, venter, and hidden surfaces such as groin, axillae, and inner faces of the hind limbs. The dorsum may present dark shades of raw umber (22) to antique brown (24) with irregular sepia (279) blotches finely outlined by whitish lime green (111), as in DHMECN 19220 (A), or lighter dorsal coloration with lower contrast, with shades ranging from cinnamon (21) and pale sulphur yellow (92), as in DHMECN 19221 (E), DHMECN 19223 (C), DHMECN 19224 (F), and DHMECN 19226 (D), to olive yellow (117). Dorsal blotches vary from dark brownish olive (127), as in DHMECN 19221 (E) and DHMECN 19222 (B), to sepia (279), as in DHMECN 19220 (A) and DHMECN 19224 (F). The interorbital, prefrontal, and eyelid regions vary from grayish olive (274), as in DHMECN 19220 (A), to dusky brown (285), as in DHMECN 19221 (E), DHMECN 19222 (B), DHMECN 19223 (C), DHMECN 19224 (F), and DHMECN 19226 (D). Canthal, labial, and supratympanic markings show colors ranging from sepia (286) and olive horn (16), as in DHMECN 19220 (A), to dark brownish olive (127) with pale sulphur yellow (92) interspaces, as in DHMECN 19221 (E), DHMECN 19222 (B), DHMECN 19223 (C), DHMECN 19224 (F), and DHMECN 19226 (D). The ventral region shows dark tones such as vandyke brown (281), with the throat in dusky brown (285) and mandibular edges pale lime green (112), as in DHMECN 19220 (A), to lighter venters such as pale sulphur yellow (92) with olive brown (278) blotches, as in DHMECN 19221 (E) and DHMECN 19224 (F), or hair brown (277), as in DHMECN 19222 (B), DHMECN 19223 (C), and DHMECN 19226 (D). The throat may be olive brown (278), as in DHMECN 19221 (E), DHMECN 19223 (C), DHMECN 19224 (F), and DHMECN 19226 (D), or vandyke brown (281), as in DHMECN 19220 (A) and DHMECN 19222 (B). The inguinal, axillary, and thigh regions may exhibit a distinctive reddish spinel pink (235), as in DHMECN 19220 (A) and DHMECN 19222 (B), or be absent, as in DHMECN 19221 (E), DHMECN 19223 (C), DHMECN 19224 (F), and DHMECN 19226 (D). The same occurs with the hidden surfaces of the legs, which may also be evident, as in DHMECN 19220 (A) and DHMECN 19222 (B), or attenuated, as in the other evaluated specimens DHMECN 19221 (E) and DHMECN 19224 (F).

**Etymology**. The specific epithet “cayapas” is used as a noun in apposition. It refers to the Cotacachi-Cayapas National Park, Piñán, Cotacachi, Imbabura Province, Ecuador, where the type series was collected.

**Figure 8:**
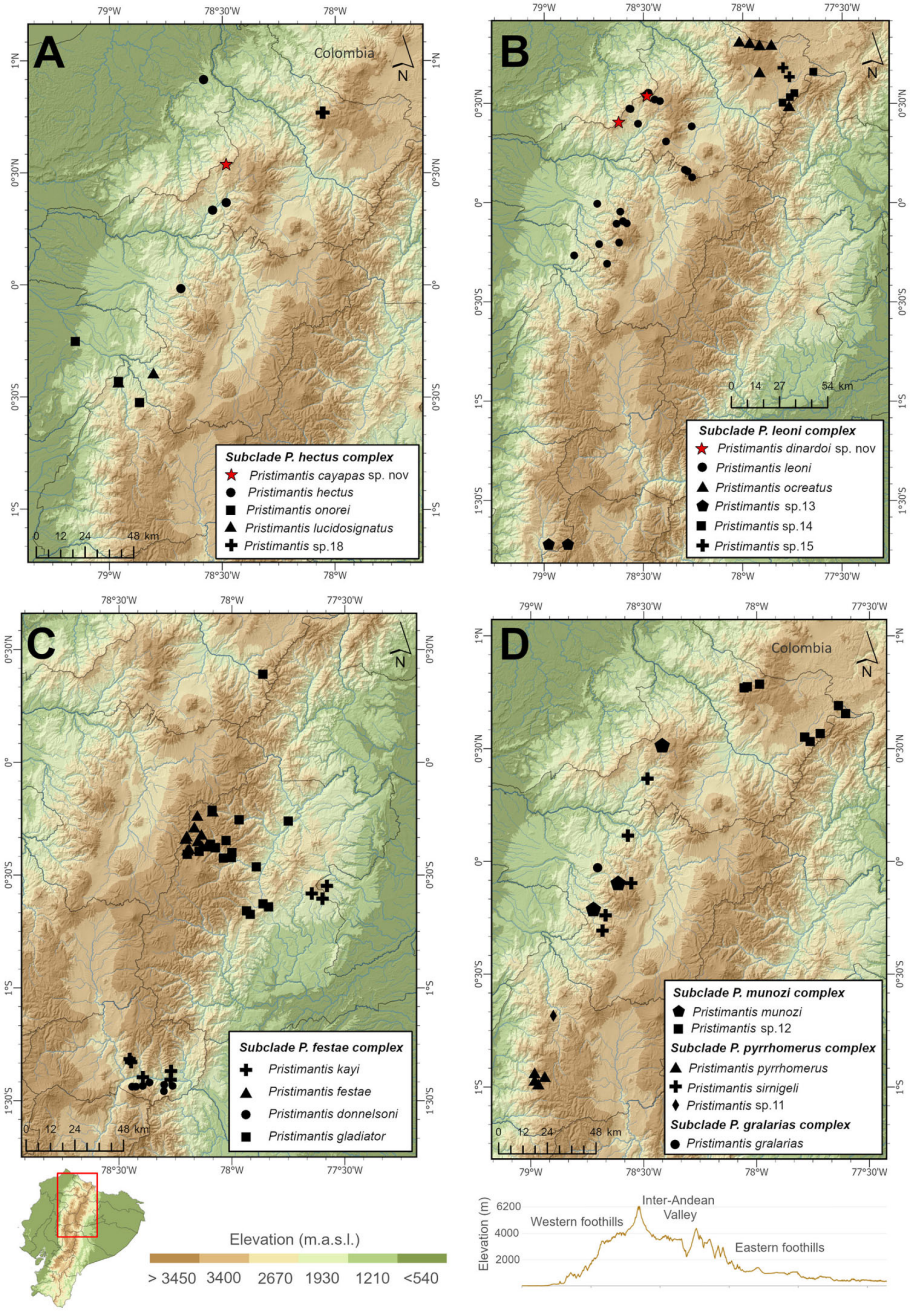
Geographic distribution of species in the *Pristimantis myersi* clade across the northern Andes of Ecuador and southern Colombia. (A) Subclade *P. hectus* complex, including P. cayapas sp. nov. (red star), *P. hectus*, *P. onorei*, P. lucidosignatus, and Pristimantis sp. 18. (B) Subclade *P. leoni* complex, including P. dinardoi sp. nov. (red star), *P. leoni*, *P. ocreatus*, and the undescribed lineages Pristimantis sp. 13, sp. 14, and sp. 15. (C) Subclade *P. festae* complex, including P. kayi, *P. festae*, P. donnelsoni, and *P. gladiator*. (D) Subclades P. munozi, *P. pyrrhomerus*, and P. gralarias complexes, including P. munozi, Pristimantis sp. 12, *P. pyrrhomerus*, Pristimantis sp. 11, P. simigei, and P. gralarias. Elevation gradient is shown below (m a.s.l.), indicating western foothills, inter-Andean valleys, and eastern foothills. Inset: geographic location of the study area within Ecuador.

**Distribution and natural history.** *Pristimantis cayapas* sp. nov. is known from the type locality in Piñan, Cotacachi, Cayapas-Chupa, Parque Nacional Cotacachi-Cayapas, Imbabura Province, Ecuador, between 3,290 to 3,410 m of elevation (Fig. 8). This species is found in the high montane evergreen forest of the Western Andes (Ministerio del Ambiente del Ecuador (MAE), 2013), in the Cayapas mountain range. This habitat is characterized by a closed canopy and trees up to 15 m high, saturated with epiphytes, bromeliads, ferns and bryophytes. Tree crowns were full of epiphytic bulbs that encourage crown falls, forming natural clearings. The seven specimens of *Pristimantis cayapas* sp. nov. were collected active at night between 20:00 and 00:00 perching herbaceous vegetation, between 10 and 150 cm high. They were found in syntopy with: *P. munozi*, *P. vertebralis*, *P. dinardoi* sp. nov., *Hyloscirtus princecharlesi* and *Gastrotheca plumbea*.

***Pristimantis dinardoi* sp. nov.**

**LSIDurn:lsid: zoobank.org:act:E4154F9C-095E-4DAE-AEB4-6A402D94719**

***Common name in Spanish:*** *Cutín de Joseph Dinardo*

***Suggested common English name:*** *Dinardo’s rainfrog*

**Figure 9:**
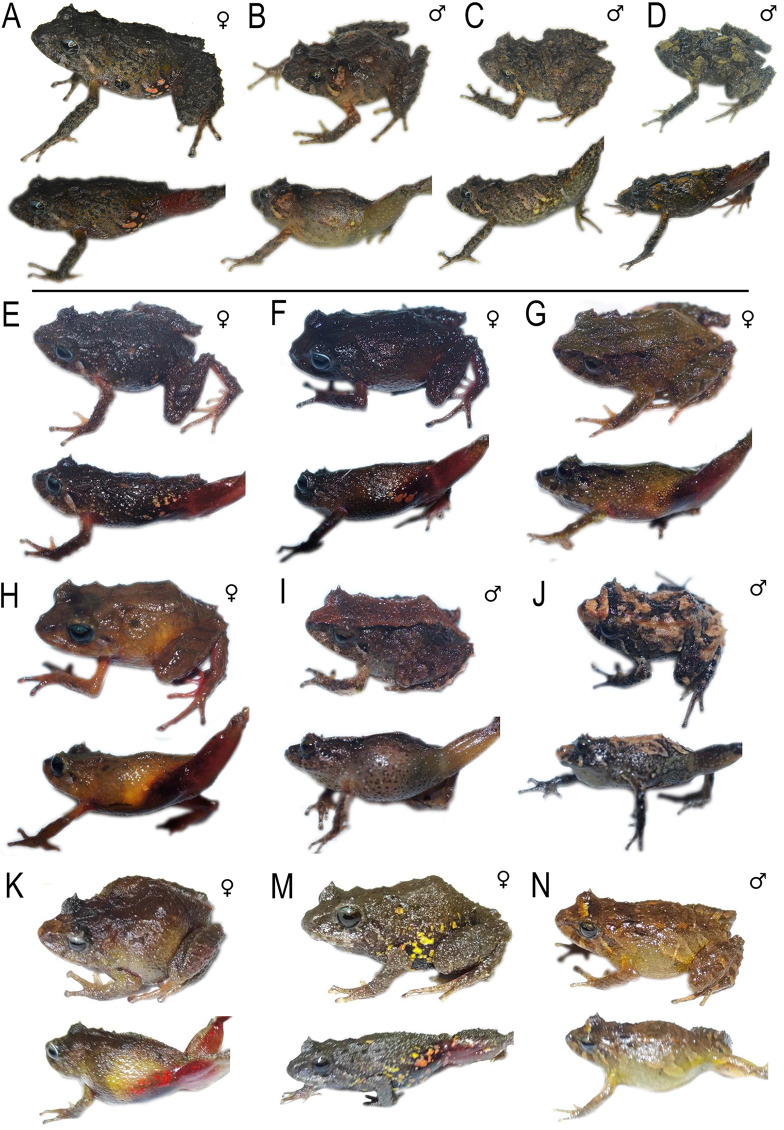
Comparison *Pristimantis* (*Trachyphrynus*) *myersi* clade from Ecuador. Subclade *P. leoni* complex: (A–D) *P. leoni*, (A) female (DHMECN 16832), (B) male (DHMECN 16831) (C) male (DHMECN 16829), (D) male (DHMECN 16828); (E–N) *P. dinardoi* sp. nov., (E) female (DHMECN19184), (F) male (DHMECN 19188), (G) female (DHMECN 19199), (H) female Holotype (DHMECN 19202), (J) male (DHMECN 19178), (I) male (DHMECN 19179), (K) male (DHMECN 18696), (L) male (DHMECN 18699), (M) male (18699), (N) male (DHMECN18736). Photographs: All photographic by Mario H. Yánez-Muñoz and Christian Paucar-Veintimillla.

**Figure 10:**
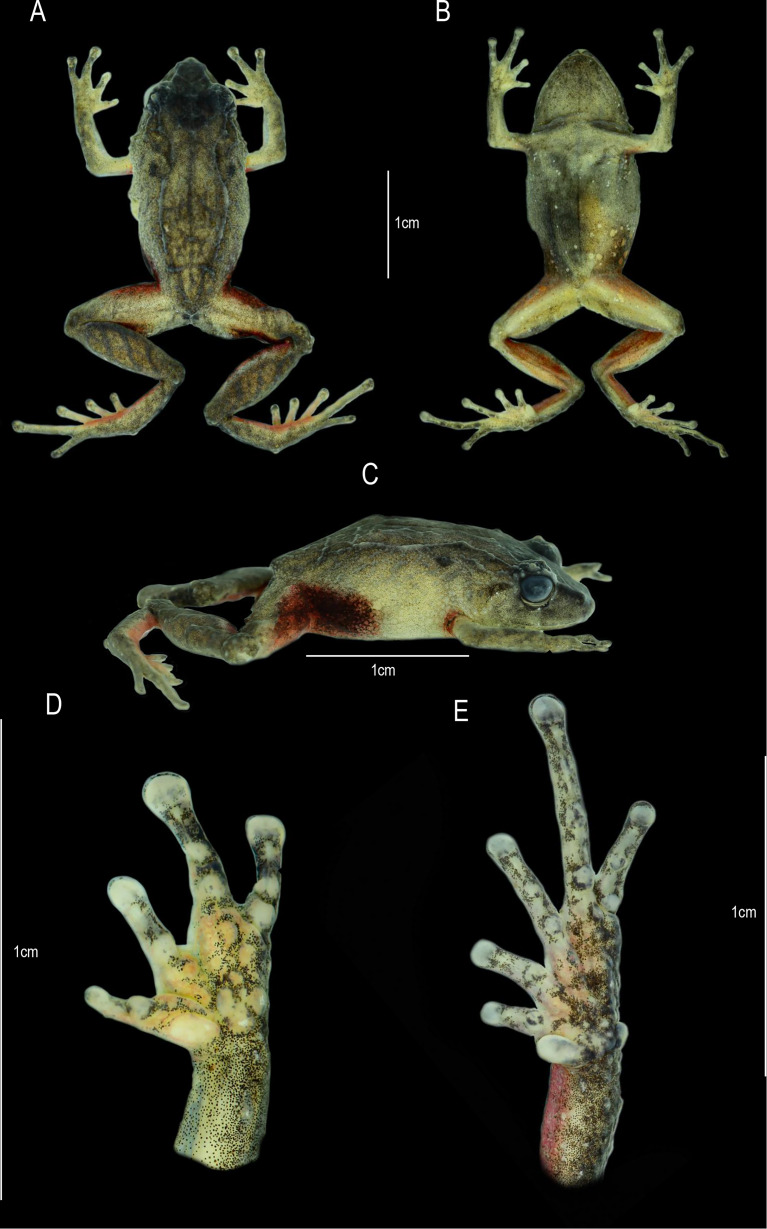
*Pristimantis dinardoi* sp. nov., holotype DHMECN 19202, adult female from Cotacachi, Imbabura Province, Ecuador. (A) Dorsal view. (B) Ventral view. (C) Lateral view of body. (D) Palmar view of right hand. (E) Plantar view of right foot. Scale bars = 1 cm. Photographs by: Katherine Nicolalde-Tapia.

**Holotype** (Figs. 2, 5, 6, 7, 9, 10). DHMECN 19202, adult female, from Cayapas Chupa, Intag canton, Cotacachi, Imbabura Province, Ecuador (0.53964, −78.48306; 3,260 m), collected on 5 September 2023 by Mario Humberto Yánez-Muñoz, Jorge Jhobany Brito Molina & Jorge Páez.

**Paratypes** (Supplemental Fig. SM1.3, Figs. 8, 9, 9♀, 8♂). From Cayapas Chupa, Intag, Cotacachi, Imbabura Province, Ecuador, with the same data as the holotype (collected by Mario Humberto Yánez-Muñoz, Jorge Jhobany Brito Molina & Jorge Páez): Adult females (5): DHMECN 19180 (0.54117, −78.48346; 3,290 m), DHMECN 19188 (0.53896, −78.48268; 3,310 m), DHMECN 19193 (0.53964, −78.48306; 3,260 m), DHMECN 19184 (0.53813, −78.48255; 3,290 m), DHMECN 19203 (0.53964, −78.48306; 3,260 m). Adult males (5): DHMECN 19178 (0.54117, −78.48346; 3,290 m), DHMECN 19181 (0.54117, −78.48346; 3,290 m), DHMECN 19190 (0.53896, −78.48268; 3,310 m), DHMECN 19201 (0.54117, −78.48346; 3,290 m), DHMECN 19204 (0.53896, −78.48268; 3,310 m). From Reserva Natural Kinti Toisán, Cotacachi, Imbabura Province, Ecuador (collected by Christian Paucar Veintimilla & Evelin Gabriela Lagla Chimba on September 17^th^ 2022): Adult females (4): DHMECN 18696, DHMECN 18697, DHMECN 18715, DHMECN 18724 (0.40674, −78.62488; 3,444 m). Adult males (3): DHMECN 18736, DHMECN 18738, DHMECN 18739 (0.40674, −78.62488; 3,444 m).

**Diagnosis.** *Pristimantis dinardoi* sp. nov. is a member of the *Pristimantis myersi* species group and clade, characterized by the following combination of characters: (1) dorsal skin shagreen with postocular, squamosal, scapular, dorsolateral and postero-sacral dermal ridges, with a pair of occipital, scapular, sub-scapular and post-sacral subconical tubercles, with short ridges scattered throughout the dorsum; venter and flanks aerolate, discoidal fold absent, dorsolateral folds absent; (2) tympanum present, tympanic annulus ¾ visible, defined, rounded, horizontal diameter of tympanum equal to 44.78% of eye diameter (*n* = 18); (3) snout short, subacuminate in dorsal view (tip pointed), and curved anteroventrally; (4) upper eyelid with one conical tubercle and two or three subconical to rounded tubercles; cranial crest absent; (5) dentigerous processes of vomers positioned posterior to the level of the choanae, slightly triangular in outline, with two to four teeth; (6) males with vocal slits, nuptial pads absent; (7) finger I shorter than finger II; discs dilated, wider than the digit, mainly in fingers III and IV, with circunmarginal grooves; (8) fingers with lateral fringes present; (9) ulnar tubercles present, with two to three subconical tubercles; (10) heel with a conical tubercle surrounded by subconical tubercles, outer edge of the tarsus bearing two to three conical tubercles; inner metatarsal fold present; (11) metatarsal tubercle oval larger (three times) than the external one which is rounded; supernumerary plantar flattened tubercles; (12) toes bearing narrow lateral fringes, basally pronounced, interdigital membrane absent, digit pads of the toes slightly wider than the digits, rounded, toe V longer than toe III, does not extend to the distal subarticular tubercle of toe IV; (13) dorsally light brown with surface of head dark brown, to homogeneously dark brown or greyish-brown. Groin, armpits and hidden surfaces of thighs and legs dark purple to red orange (in life from dark bluish-purple, salmon or bright red, bright yellow). Ventrally from grayish-cream to brownish gray; (14) females SVL= 17.26–25.88 mm (mean = 20.55 mm, *n* = 10); males SVL = 13.09–16.06 mm (mean = 14.83 mm, *n* = 8).

**Comparison with other species (**Figs. 2–5, 9, Supplemental Figs. SM1.3–SM1.4**).** The new species is closely related to several lineages within the *P. leoni* complex, including three undescribed taxa (Fig. 1). However, it differs from the formally described members of the group, *P. leoni* and *P. ocreatus*, in the following diagnostic characters: body size (SVL = 20.55 mm in females and 14.83 mm in males), tympanum present, digital pads of fingers wider than the digits and in toes slightly wider than the digits, and heel with a conical tubercle surrounded by subconical tubercles.

*Pristimantis dinardoi* is most similar to *P. leoni* Lynch, 1976, mainly based on presence of red coloration on the groin and dermal ridges. However, it can be distinguished by the tympanum being present and externally visible (partially concealed beneath the skin in *P. leoni*) and by its smaller size (SVL = 20.55 mm in adult females, *n* = 10, *vs*. 21.9 mm in adult females of *P. leoni*, *n* = 23). In *P. ocreatus* Lynch, 1981 the tympanum is completely concealed, whereas in *P. dinardoi* it is visible. Furthermore, *P. ocreatus* lacks enlarged or distinctive tubercles on the heel and tarsus, while *P. dinardoi* bears a conical heel tubercle surrounded by subconical tubercles. Finally, the digital pads of the fingers are as wide as the digits in *P. ocreatus* (Lynch, 1981), whereas they are distinctly wider than the digits in *P. dinardoi* (Fig. 9).

Genetic distances between *Pristimantis dinardoi* and its closely related species (*P. leoni*, *P*. sp. 13, *P*. sp. 14, *P. ocreatus*, and *P*. sp. 15) are 2.61%, 2.56%, 4.75%, 4.62%, and 6.39%, respectively. Genetic distances with other species in the clade range from 3.44% to 6.77% (Supplemental Fig. SM1.1).

**Description of the holotype (**Figs. 5, 10**).** Adult female (DHMECN 19202), head longer than wide. Snout short, curved anteroventrally in profile, subacuminate in dorsal view, tip pointed; eye-nostril distance 10.03% of SVL, canthus rostralis in cross section rounded, in dorsal view slightly concave, loreal region concave, protruding nerines and directed laterally; interorbital area slightly flat, interorbital distance wider than upper eyelid, 84.73%; cranial crest absent, with a perpendicular postocular ridge extending towards the occipital tubercle, with squamosal ridges extending beyond the scapular tubercles, with “) (“ shaped scapular ridges, with dorsolateral and sacral ridges, a pair of subscapular subconical tubercles; cranial crest absent, tympanum present, tympanic annulus ¾ visible, anteriorly defined, rounded, slightly visible dorsally, horizontal diameter of tympanum equal to 44.78% of eye diameter, supratympanic margin formed by a row of four subconical tubercles and a thin supratympanic fold; postrictal tubercles present, two large subconical surrounded by several flattened tubercles; choanae small, rounded, not covered by palatal floor of maxilla; dentigerous processes of vomers positioned posterior to the level of the choanae, slightly triangular in outline, with three or four teeth, tongue oval as long as wide, 30% attached to palatal floor of the mouth.

Texture of dorsum dorsal skin shagreen with postocular, squamosal, scapular, dorsolateral and postero-sacral dermal ridges, with a pair of occipital, scapular, sub-scapular, antero-sacral and post-sacral subconical tubercles, venter and flanks aereolate; discoidal fold absent; two subconical ulnar tubercles present; outer palmar trilobed tubercle “U”-shaped, slightly smaller than the ovoid outer thenar tubercle; several supernumerary tubercles, rounded; subarticular tubercles defined, rounded in lateral view. finger I shorter than finger II; discs dilated, fingers with lateral fringes present, basally pronounced, slightly wider than the digit, mainly in fingers III and IV, with circunmarginal grooves. Hind limbs slender (TL 48.65% of SVL; FL 46.72% of SVL) heel with a conical tubercle surrounded by subconical tubercles, outer edge of the tarsus bearing two to three conical tubercles; inner metatarsal fold present; two metatarsal tubercles, external small subconical, internal ovoid 4X larger than external tubercle; supernumerary plantar flattened tubercles; toes with defined lateral fringes, basally pronounced, interdigital membrane absent. Digits and discs narrow, toe V longer than toe III, does not extend to the distal subarticular tubercle of the IV toe.

**Holotype coloration in preservative (**Fig. 10**).** Dorsal coloration olive horn (16) with greyish olive spots (274). Cephalic region, subscapular and post-sacral tubercles, as well as canthal region and supratympanic bar hooker’s green (138). Squamosal, scapular, dorsolateral and postero-sacral dermal ridges in color paris white (139). Dorsal surfaces of legs with greyish olive bars (274) with pale sulphur yellow interspaces (92). Flanks pale sulphur yellow (92), axillae and groin markings in range of mahogany red (34) to maroon colour (39). Ventrally in glaucous ground (289) with central region sulphur yellow (80) finely dotted with grey, chest and throat whitish lime green (111) finely dotted with grey. Rear surfaces of the thighs mahogany red (34), dorsal surfaces at the base of toes I–IV salmon colour (251). Hidden surfaces of the legs peach red (70).

**Holotype coloration in life (**Fig. 5**).** Dorsum true cinnamon (260) with inteorbital region, canthal, supratympanic and subscapular tubercles vandyke brown (282). Flanks in dorsolateral and middle anterior region buff yellow (6), middle posterior region light orange yellow (77). Dorsal surfaces of the digits of the foot, hidden surfaces of axillae, groin, legs and thighs geranium pink (241). Groin dark carmine (61) in the central region, with outer edges in range from gem ruby (65) to geranium pink (241). Belly in the upper region amber (22), in lower region crimson (62), ventral surfaces of thighs and feet geranium pink (242). Throat salmon colored (58). Iris paris white (139) with fine black reticulations, with middle band dusky brown (285) with white sclera.

**Measurements (in mm) of holotype.** SVL = 20.12; HW = 7.23; HL = 7.68; ED = 2.68; IOD = 2.30; EN = 2.02; TD = 1.20; TL = 9.79; EW = 1.72; FoL = 9.40; HaL = 4.98; FW = 0.79; TW = 0.78.

**Variation.** Morphometric variation in the type series is shown in Supplemental Table SM2.1 Material S3 and Supplemental Fig. S1.2. The coloration in life of *Pristimantis dinardoi* (Fig. 9) shows marked variation. On the dorsum, the holotype exhibits a true cinnamon (260) tone, whereas other individuals display colors such as fuscous (283) DHMECN 19184 (E), warm sepia (40) DHMECN 19188 (F), grayish olive (274) DHMECN 19199 (G), or combinations such as dark mauve (208) and ferruginous (35) DHMECN 19179 (I). In the interorbital bar, the holotype is vandyke brown (282), contrasting with darker tones such as dusky brown (285) DHMECN 19178 and 18696 (J, K) or dark carmine (61) DHMECN 18736 (N). The flanks also show high chromatic diversity: the holotype presents a mixture of buff yellow (6) with light orange yellow (77), whereas other specimens present dusky brown (285), as in DHMECN 19184, 19188, 19199, and 18696 (E, F, G, K), light bluish purple (228) DHMECN 19179 (I), or even greyish and orange blotches, as in DHMECN 18699 (M). On the hidden surfaces of the limbs, the holotype shows geranium pink (241), a color also observed in DHMECN 18696 (K), while other individuals such as DHMECN 19184 and 19188 (E, F) present tones like smoky red, orange tint DHMECN 19199 (G), or smoky white (261) DHMECN 19179 and 19178 (I, J). In the inguinal region, the holotype exhibits a transition from dark carmine (61) and gem ruby (65) to geranium pink (241), whereas other specimens show medium purple (226) DHMECN 19179 (I), bluish purple (229) DHMECN 19178 (J), or simply “pinkish” DHMECN 19199 (G). Ventral coloration varies from the pattern of the holotype (amber (22) and crimson (62)) to darker tones such as dusky brown (285) DHMECN 19184, 19188, 19199, 19178, and 18696 (E, F, G, J, K), blue black (187) DHMECN 19179 (I), or greyish patterns with marks, as in DHMECN 18699 (M). Finally, the iris is consistent among individuals, with a base of paris white (139), black reticulation, and a dusky brown (285) band in all specimens.

In preservative (Supplemental Fig. SM1.3), the specimens examined show notable variation in preserved colouration. The holotype (DHMECN 19202) displays a dorsum of olive horn (16) with grayish olive (274) blotches, in contrast to paratypes that exhibit variations towards greyish tones, as in DHMECN 19184 (B), DHMECN 19179 (I), DHMECN 19178 (E), olive (126) to dark brownish (127) DHMECN 19188 (C), DHMECN 19199 (D), DHMECN 18699 (G), or pale lime green (112) DHMECN 18696 (F), DHMECN 18736 (H). The cephalic region shows a defined pattern of hooker’s green (138), shared with some specimens (DHMECN 19188 (C), DHMECN 19178 (E), DHMECN 18699 (G), DHMECN 18736 (H)), whereas others present sepia (286) (DHMECN 19184 (B), DHMECN 18696 (F), DHMECN 18736 (H, partial)) or combinations with darker greys (DHMECN 19199 (D), DHMECN 19179 (I)). The subscapular and postsacral tubercles are also hooker’s green (138), contrasting with the predominance of cyan white (155) in most paratypes, except DHMECN 19188 (C), which shows medium neutral gray (298), and DHMECN 18696 (F), which combines sepia and cyan white. Dermal ridges are paris white (139) in the holotype, a pattern also found in DHMECN 19178 (E) and DHMECN 18696 (F), while other specimens alternate with tones such as light sky blue (191) DHMECN 19184 (B), medium neutral gray (298) DHMECN 19188 (C), cyan white DHMECN 19199 (D), DHMECN 19179 (I), or greenish combinations in DHMECN 18699 (G) and DHMECN 18736 (H). The dorsal surfaces of the legs combine grayish olive (274) with pale sulphur yellow (92), differing from variations towards olive (126) to dark brownish (127) in DHMECN 19188 (C), DHMECN 19199 (D), DHMECN 19179 (I), DHMECN 18699 (G), or pale lime green (112) with dark marks in DHMECN 19178 (E), DHMECN 18696 (F), DHMECN 18736 (H). The flanks are pale sulphur yellow (92), contrasting with darker tones in some specimens (DHMECN 19184 (B), DHMECN 19179 (I), DHMECN 19178 (E)) or greenish hues in others (DHMECN 19199 (D), DHMECN 18696 (F), DHMECN 18736 (H)). In the axillae and groin, the holotype exhibits an intense reddish tint (mahogany red to maroon), compared with pinkish tones such as pinkish flesh color (253) (DHMECN 19184 (B), DHMECN 19188 (C), DHMECN 18696 (F), DHMECN 18699 (G)), or absence of marks (DHMECN 19199 (D), DHMECN 19179 (I), DHMECN 19178 (E), DHMECN 18736 (H)). Ventrally, the holotype combines glaucous (289) with a central region of sulphur yellow (80) dotted with grey, a distinctive feature compared with the predominance of pale lime green (112) with brown or grey blotches in the others. On the chest and throat, a whitish lime green (111) dotted with grey is observed, lighter than the greenish pale lime green (112) with brown or grey spotting of the paratypes. Likewise, the posterior surfaces of the thighs are mahogany red (34) in the holotype, contrasting with pinkish flesh color (253) or salmon color (251) in most paratypes, while the base of the fingers is salmon color (251), compared with pinkish or greenish tones in the other specimens. Finally, the hidden surfaces of the legs are a peach red (70), contrasting with the predominance of pinkish flesh color (253) or pale lime green (112) with blotches in the remainder of the series.

**Etymology.** The specific epithet “dinardoi”, it is an honor to name this new species in recognition of Mr. Joseph Rex Dinardo Jr., a passionate naturalist and bibliophile specializing in amphibians and reptiles. His legacy encompasses nearly one hundred thousand books and publications that now form the basis of the herpetological library of the Center for Research on Amphibian and Reptile Diversity at the University of Texas at Arlington and the biodiversity library of the National Institute of Biodiversity of Ecuador.

**Distribution and natural history.** *Pristimantis dinardoi* sp. nov. is known only from two localities: the type locality at Cayapas Chupa, Intag, Cotacachi, Imbabura, Ecuador, and Reserva Natural Kinti Toisán, Cotacachi, Imbabura, Ecuador, at elevations between 3,260 and 3,444 m a.s.l. The new species inhabits high montane evergreen forests of the Western Andes (Ministerio del Ambiente del Ecuador (MAE), 2013), within the Cayapas mountain range. This habitat is characterized by a dense canopy up to 15 m tall, with trees heavily covered in epiphytes, bromeliads, ferns, and bryophytes. Individuals were collected at night, between 7:42 and 9:00 p.m., found either among leaves of bushes 10–40 cm above the ground, or in moss close to the ground. *Pristimantis dinardoi* sp. nov. was found in syntopy with species such as *P. munozi*, *P*. sp., and *P. cayapas*.

## Discussion

This study updates the species limits of the *Pristimantis myersi* clade within the *P. myersi* species group, which also includes the *P. jubatus*, *P. celator*, and *P. myersi* clades (Yánez-Muñoz et al., 2025). Integrating morphological, molecular, and geographical evidence, our results complement and extend the phylogenetic inferences of previous works such as Franco-Mena et al. (2023) and Yánez-Muñoz et al. (2025), who documented the clade’s diversification, high phylogenetic diversity, and the biogeographic significance in the western Andean slopes. We describe two new species (*P. cayapas* and *P. dinardoi*) and confirm at least six new candidate species, all with restricted distributions within the northern slopes of the Ecuadorian Andes (Figs. 1, 8).

For the recognition of these candidate species, we adopted a conservative integrative criterion combining phylogenetic and morphological evidence. We considered unnamed lineages as candidate species when they (1) formed highly supported monophyletic clades in our analyses (typically ≥ 80 SH-aLRT and ≥ 95 bootstrap), (2) were congruent with the clades recovered in Franco-Mena et al. (2023) and Yánez-Muñoz et al. (2025), and (3) showed consistent morphological differences relative to closely related, formally described species. Although these lineages likely represent undescribed species, their formal taxonomic treatment is beyond the scope of this study.

The type locality of *Pristimantis munozi* is Reserva Verdecocha, Pichincha Province, Ecuador, at an elevation of 2,851 m (Rojas-Runjaic, Delgado & Guayasamin, 2014). *Pristimantis floridus* was originally described from its type locality in Sigchos, Cotopaxi, with additional paratypes referred from Pichincha and Imbabura, all populations characterized by expanded digital pads in both sexes (Lynch & Duellman, 1997). Later, Rojas-Runjaic, Delgado & Guayasamin (2014) described *P. munozi* based on male specimens from Pichincha populations, providing a phylogenetic placement for the species but without including material of *P. floridus*. To date, no phylogenetic placement has been established for *P. floridus*. In this work, we present new records of *P. munozi* that extend its known distribution by approximately 70 km. The genetic distance between topotypic populations and these newly recorded localities is 1.3%, and their morphology is consistent. Our results indicate that populations from Pichincha and Imbabura attributed to *P. munozi* show no significant genetic divergence. Consequently, we suggest that the description by Lynch & Duellman (1997) of *P. floridus* may represent a broadly distributed lineage ranging from Cotopaxi to Imbabura (Fig. 8). We note that although some lineages may show genetic distances slightly below 2%, their morphological differentiation and spatial distribution are congruent (*e.g*., *P. gladiator*, *P. kayi* and *P. donnelsoni*; Reyes-Puig et al. (2023). By contrast, lineages with genetic distances below 1.5% often present greater challenges in terms of morphological and ecological niche differentiation. Therefore, we suspect that both populations belong to *P. floridus*.

We observed clear genetic differentiation with closely related species in some taxa, such as *P. cayapas* (>4% *p*-distance) and *P. dinardoi* (>2.5% *p*-distance), exceeding or approaching commonly used thresholds for species delimitation in Neotropical frogs (typically 2–3%; Fouquet et al., 2007; Funk, Caminer & Ron, 2012). By contrast, other closely related species, such as *P. onorei* and *P. lucidosignatus* (1.3% genetic distance), exhibit lower molecular divergence. A similar situation is found in *P. donnelsolni* and *P. gladiator* (1.8% genetic distance). In the former, morphological and geographical limits remain unclear (type locality Tandapi; Rodder & Schmitz, 2009), as both species originate from the same locality, whereas in the latter case, morphological and ecological differences are congruent (Reyes-Puig et al., 2023). These examples highlight the need to integrate multiple lines of evidence (*e.g*., morphological traits, biogeographical patterns, and, when available, bioacoustic data) rather than relying solely on genetic distances to delimit species. Such an integrative approach is essential to resolve taxonomic and phylogenetic puzzles among related species, particularly in groups that have undergone recent diversification (Vieites et al., 2009).

## Conclusions

Our work describes two new species of the *Pristimantis myersi* clade from the western Andes of Ecuador. We provide evidence for their distinction based on morphological and genetic divergence. Our results expand the diversity of this clade in the northern Andes and contribute to clarifying its phylogenetic relationships. Integrating morphological and molecular evidence, we hypothesize about the boundaries between *P. onorei* and *P. lucidosignatus*, as well as between *P. munozi* and *P. floridus*, reinforcing the need for a comprehensive approach to resolve the taxonomic and phylogenetic complexity within the group.

## Supplemental Information

10.7717/peerj.21075/supp-1 Supplemental Information 1 Figures of Genetic distance matriz and variation of two new species of Pristimantis of the *P. myersi* clade. Fig. SM1.1. Pairwise genetic distances (uncorrected p-distances, in %) among species of the *Pristimantis myersi* clade, based on mitochondrial DNA sequences (16S). The heatmap shows the percentage divergence between species pairs, with values color-coded according to the scale on the left. The dendrogram represents phylogenetic relationships inferred from the same dataset. The two new species described herein, *Pristimantis cayapas* sp. nov. and *P. dinardoi* sp. nov., are highlighted in blue and red, respectively.
Fig. SM1.2. Dorsal and ventral views of Pristimantis cayapas sp. nov. from the western Andes of Ecuador. (A) DHMECN 19220; (B) DHMECN 19222; (C) DHMECN 19223; (D) DHMECN 19226; (E) DHMECN 19221; (F) DHMECN 19224. Scale bar = 1 cm. Fig. SM1.3. Dorsal and ventral views of Pristimantis dinardoi sp. nov. from the western Andes of Ecuador. (A) DHMECN 19202; (B) DHMECN 19184; (C) DHMECN 19188; (D) DHMECN 19199; (E) DHMECN 19178; (F) DHMECN 18696; (G) DHMECN 18699; (H) DHMECN 18736; (I) DHMECN 19179. Scale bar = 1 cm.

10.7717/peerj.21075/supp-2 Supplemental Information 2 Morphometric measurements (mean ± standard deviation, in mm) of Pristimantis cayapas sp. nov. and Pristimantis dinardoi sp. nov. by sex. Abbreviations: SVL = snout–vent length; HW = head width; HL = head length; EN = eye–nostril distance; IND = internarial distance; IOD = interorbital distance; EW = eye width; TD = tympanum diameter; ED = eye diameter; TL = tibia length; HaL = hand length; FoL = foot length; FW = finger width; TW = toe width.

10.7717/peerj.21075/supp-3 Supplemental Information 3 Examined material. Used in the description and comparisons of two new species of Pristimantis of the *P. myersi* clade.

10.7717/peerj.21075/supp-4 Supplemental Information 4 ARRIVE checklist.

## Additional Information and Declarations

### Field Study Permissions

The following information was supplied relating to field study approvals (*i.e*., approving body and any reference numbers):

The Ministry of Environment, Water, and Ecological Transition of Ecuador (MAATE) approved the study (Permit Nos. MAATE-ARSFC-2023-3346 and MAATE-ARSFC-2024-0847).

### Data Availability

The following information was supplied regarding data availability:

New sequences are available at GenBank: PX254075–PX254109.

### New Species Registration

The following information was supplied regarding the registration of a newly described species:

Publication LSID: urn:lsid:zoobank.org:pub:8E8B82A4-3803-40D9-9CF4-9BB8C66B4B51

Pristimantis cayapas sp. nov.

LSID: urn:lsid:zoobank.org:act:82585CA0-70C5-49DA-81ED-125677E57A9F

Pristimantis dinardoi sp. nov.

LSID: urn:lsid: zoobank.org:act:E4154F9C-095E-4DAE-AEB4-6A402D947193.
