# Supplementary material for: Update of species limits in the Pristimantis myersi clade (Anura: Craugastoridae), with the description of two new species from the western Andes of Ecuador
Source: PeerJ. 2026 Apr 14;14:e21075. doi: 10.7717/peerj.21075 (PMC13089221; doi:10.7717/peerj.21075)
Supplement: Supplemental Information 1 — Fig. SM1.1. Pairwise genetic distances (uncorrected p-distances, in %) among species of the Pristimantis myersi clade, based on mitochondrial DNA sequences (16S). The heatmap shows the percentage divergence between species pairs, with values color-coded according to the scale on the left. The dendrogram represents phylogenetic relationships inferred from the same dataset. The two new species described herein, Pristimantis cayapas sp. nov. and P. dinardoi sp. nov., are highlighted in blue and red, respectively. Fig. SM1.2. Dorsal and ventral views of Pristimantis cayapas sp. nov. from the western Andes of Ecuador. (A) DHMECN 19220; (B) DHMECN 19222; (C) DHMECN 19223; (D) DHMECN 19226; (E) DHMECN 19221; (F) DHMECN 19224. Scale bar = 1 cm. Fig. SM1.3. Dorsal and ventral views of Pristimantis dinardoi sp. nov. from the western Andes of Ecuador. (A) DHMECN 19202; (B) DHMECN 19184; (C) DHMECN 19188; (D) DHMECN 19199; (E) DHMECN 19178; (F) DHMECN 18696; (G) DHMECN 18699; (H) DHMECN 18736; (I) DHMECN 19179. Scale bar = 1 cm. [file peerj-14-21075-s001.docx]

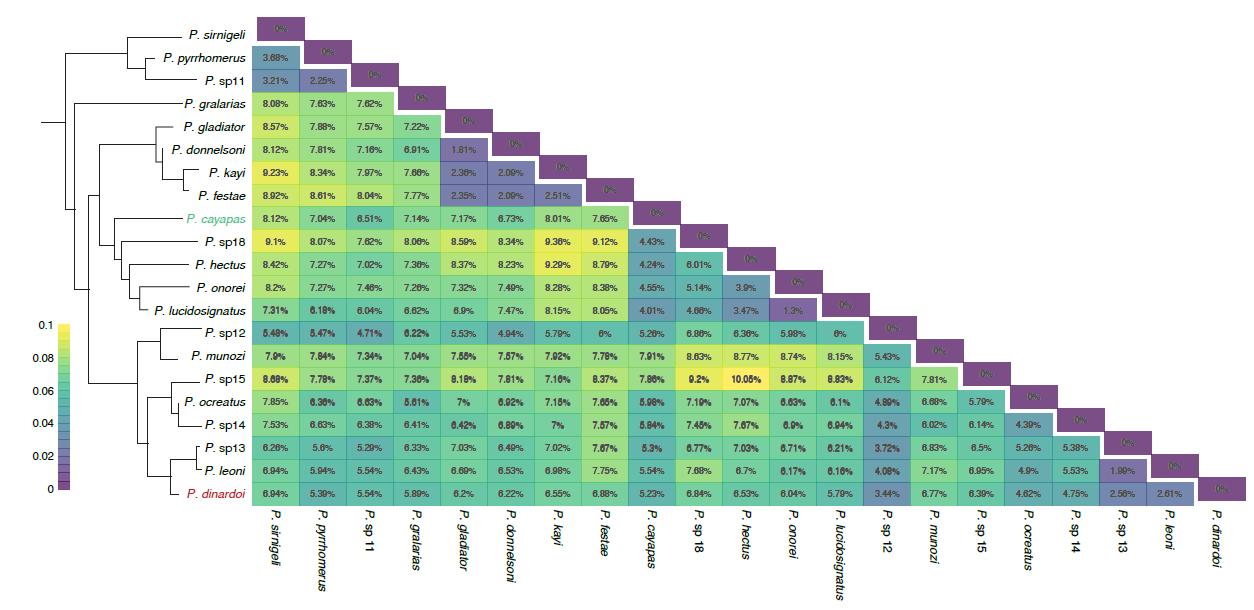


**Fig SM1.1.** Pairwise genetic distances (uncorrected p-distances, in %) among species of the *Pristimantis myersi* clade, based on mitochondrial DNA sequences (16S). The heatmap shows the percentage divergence between species pairs, with values color-coded according to the scale on the left. The dendrogram represents phylogenetic relationships inferred from the same dataset. The two new species described herein, *Pristimantis cayapas* sp. nov. and *P. dinardoi* sp. nov., are highlighted in blue and red, respectively.


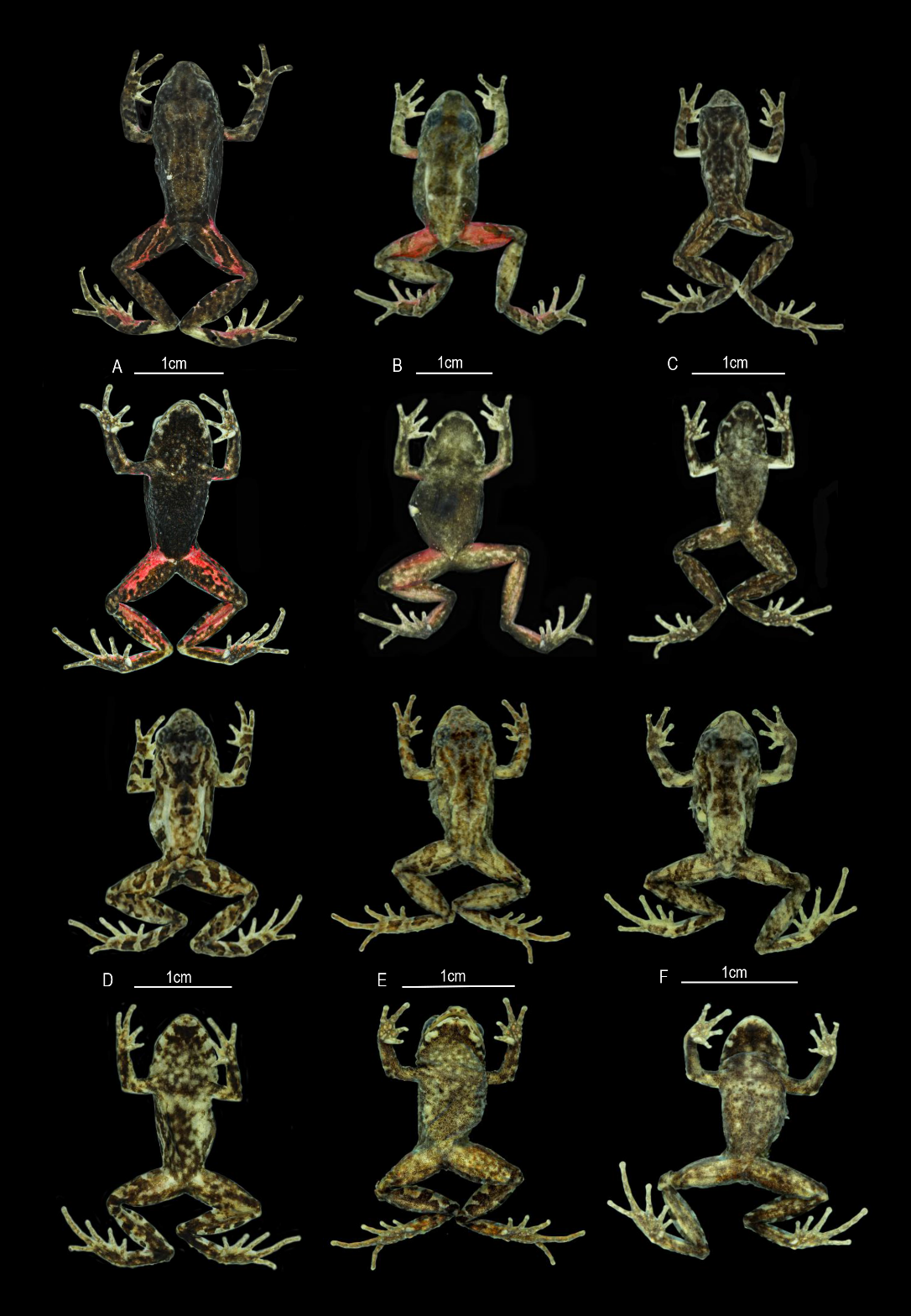


**Fig. SM1.2.** Dorsal and ventral views of Pristimantis cayapas sp. nov. from the western Andes of Ecuador. (A) DHMECN 19220; (B) DHMECN 19222; (C) DHMECN 19223; (D) DHMECN 19226; (E) DHMECN 19221; (F) DHMECN 19224. Scale bar = 1 cm.


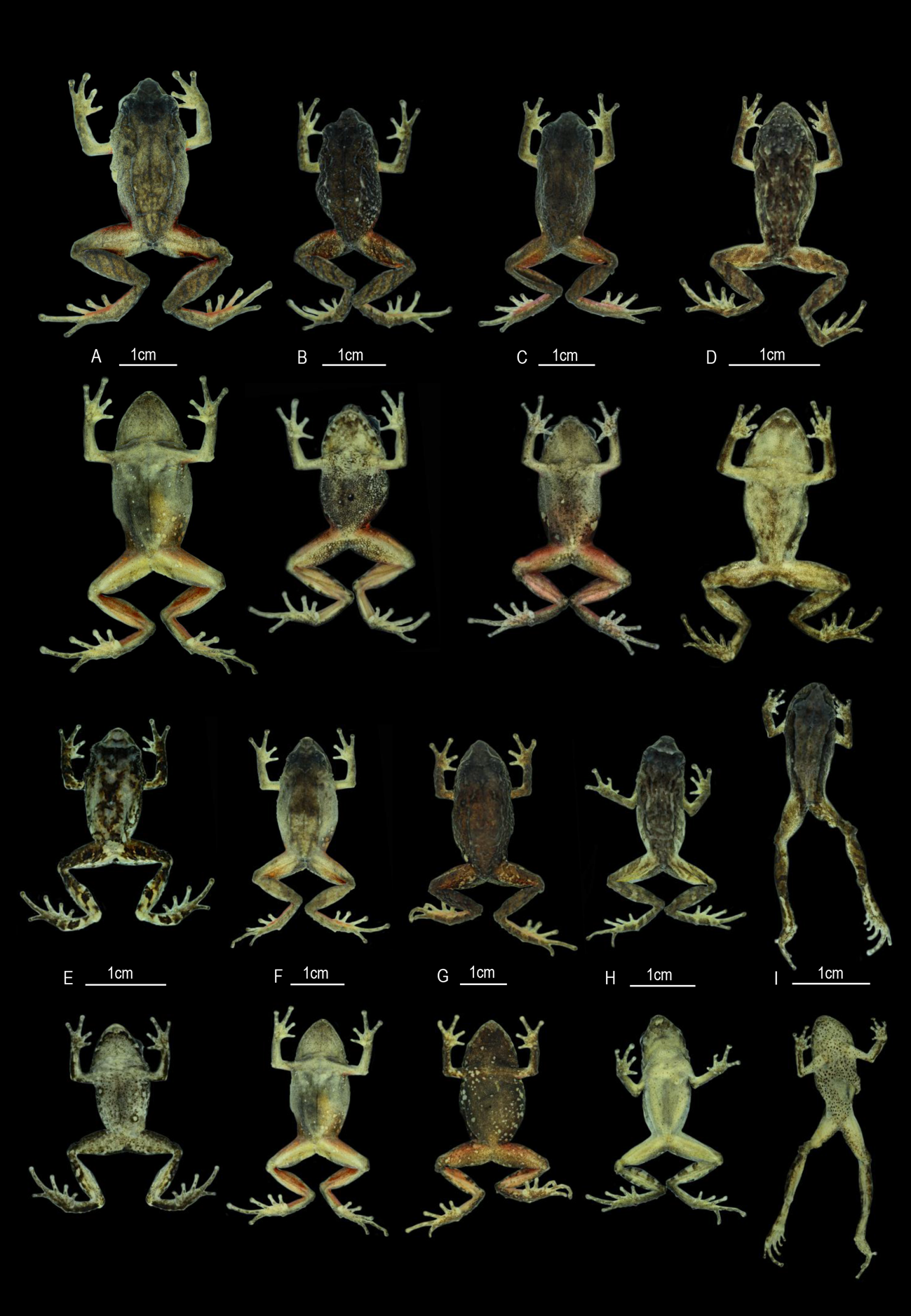


**Fig. SM1.3.** Dorsal and ventral views of Pristimantis dinardoi sp. nov. from the western Andes of Ecuador. (A) DHMECN 19202; (B) DHMECN 19184; (C) DHMECN 19188; (D) DHMECN 19199; (E) DHMECN 19178; (F) DHMECN 18696; (G) DHMECN 18699; (H) DHMECN 18736; (I) DHMECN 19179. Scale bar = 1 cm.


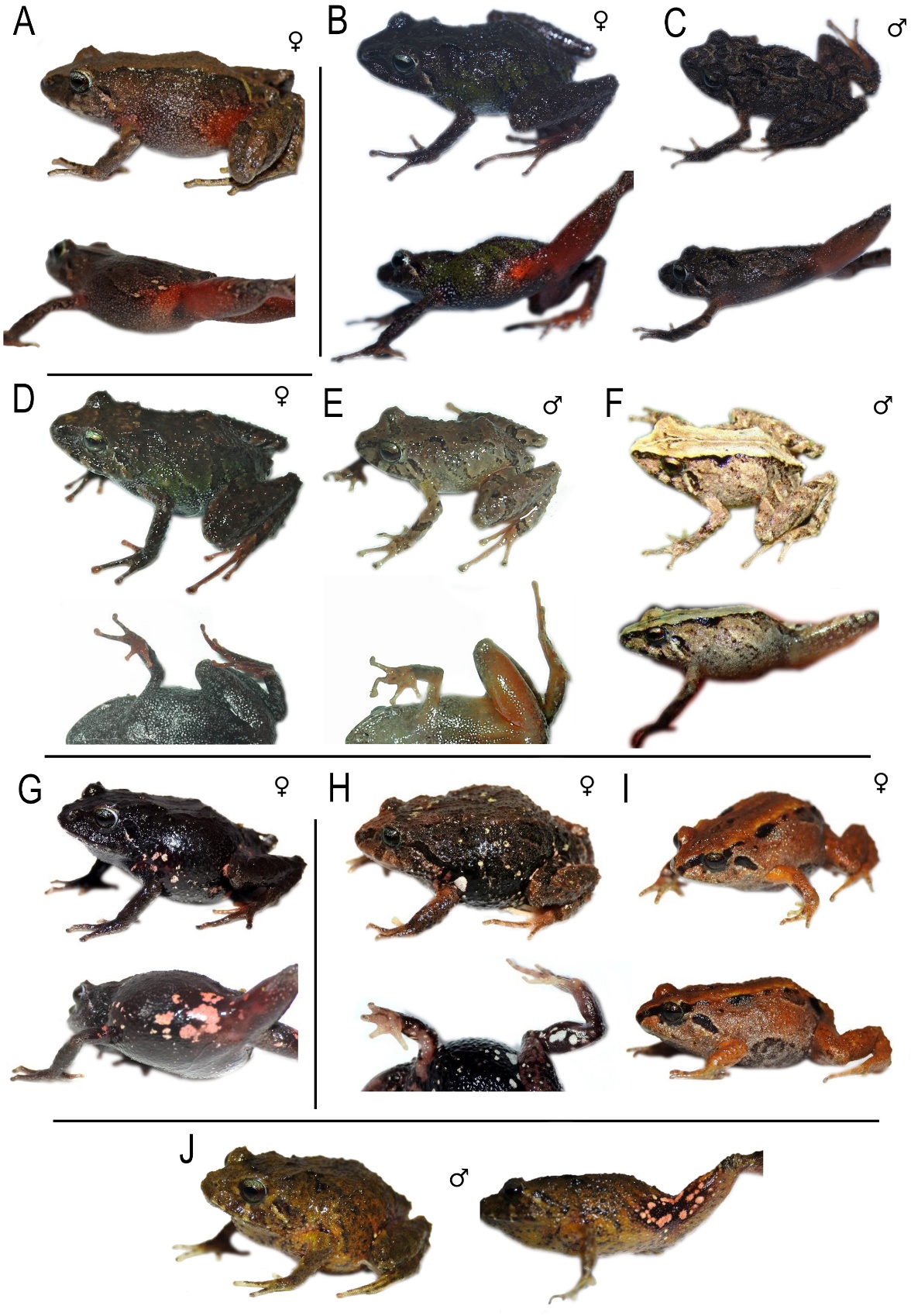


**Fig. SM1.4**. Comparison *Pristimantis* (*Trachyphrynus*) *myersi* clade from Ecuador. Subclade *P. floridus* complex: (A) *P.* sp.12, female (DHMECN 13647); (B–F) *P.* *munozi*, (B) female (DHMECN 19213, (C) male (DHMECN 19216) (D) female (DHMECN4938), (E) male (DHMECN 04944), (F) male no collected. Subclade *P.* *leoni* complex: (G) *P.* sp. 15, female (DHMECN 13635); (H) *P. ocreatus*, female (DHMECN 13655), male (DHMECN 13650); (J) *P.* sp. 14, female (DHMECN 13644). All photographic by Mario H. Yánez-Muñoz, except: (F) Fernando Rojas (G, J) Diego Batallas-Revelo.
