# Supplementary material for: Update of species limits in the Pristimantis myersi clade (Anura: Craugastoridae), with the description of two new species from the western Andes of Ecuador
Source: PeerJ. 2026 Apr 14;14:e21075. doi: 10.7717/peerj.21075 (PMC13089221; doi:10.7717/peerj.21075)
Supplement: Supplemental Information 2 — Abbreviations: SVL = snout–vent length; HW = head width; HL = head length; EN = eye–nostril distance; IND = internarial distance; IOD = interorbital distance; EW = eye width; TD = tympanum diameter; ED = eye diameter; TL = tibia length; HaL = hand length; FoL = foot length; FW = finger width; TW = toe width. [file peerj-14-21075-s002.docx]

|  | *Pristimantis cayapas* |  | *Pristimantis dinardoi* |  |
| --- | --- | --- | --- | --- |
|  | Males | Females | Males | Females |
| SVL | 13.31 ± 0.58 | 17.75 ± 0.65 | 14.83 ± 1.92 | 20.55 ± 2.72 |
| HW | 4.91 ± 0.33 | 6.38 ± 0.12 | 5.59 ± 0.39 | 7.59 ± 0.84 |
| HL | 5.16 ± 0.2 | 6.48 ± 0.07 | 5.83 ± 0.41 | 7.82 ± 0.81 |
| EN | 1.26 ± 0.09 | 1.76 ± 0.26 | 1.51 ± 0.22 | 2.07 ± 0.19 |
| IND | 1.42 ± 0.09 | 1.76 ± 0.01 | 1.67 ± 0.21 | 2.18 ± 0.25 |
| IOD | 1.75 ± 0.11 | 2.06 ± 0.14 | 1.96 ± 0.1 | 2.47 ± 0.29 |
| EW | 1.08 ± 0.1 | 1.23 ± 0.11 | 1.29 ± 0.18 | 1.67 ± 0.2 |
| TD | 0.86 ± 0.12 | 0.99 ± 0.13 | 1.05 ± 0.13 | 1.2 ± 0.11 |
| ED | 1.78 ± 0.12 | 2.16 ± 0.08 | 1.97 ± 0.17 | 2.43 ± 0.25 |
| TL | 7.02 ± 0.2 | 9.07 ± 0.25 | 7.09 ± 0.62 | 9.77 ± 0.78 |
| HaL | 3.81 ± 0.16 | 5.26 ± 0.3 | 3.69 ± 0.51 | 5.43 ± 0.58 |
| FoL | 6.88 ± 0.1 | 9.1 ± 0.47 | 6.66 ± 0.55 | 9.6 ± 1.12 |
| FW | 0.49 ± 0.07 | 0.62 ± 0.01 | 0.47 ± 0.11 | 0.76 ± 0.09 |
| TW | 0.49 ± 0.07 | 0.62 ± 0.04 | 0.47 ± 0.08 | 0.76 ± 0.11 |

**Table SM2.1.** Morphometric measurements (mean ± standard deviation, in mm) of Pristimantis cayapas sp. nov. and Pristimantis dinardoi sp. nov. by sex. Abbreviations: SVL = snout–vent length; HW = head width; HL = head length; EN = eye–nostril distance; IND = internarial distance; IOD = interorbital distance; EW = eye width; TD = tympanum diameter; ED = eye diameter; TL = tibia length; HaL = hand length; FoL = foot length; FW = finger width; TW = toe width.
